# Single-cell RNA sequencing reveals the potential mechanism of heterogeneity of immunomodulatory properties of foreskin and umbilical cord mesenchymal stromal cells

**DOI:** 10.1186/s13578-022-00848-w

**Published:** 2022-07-22

**Authors:** Siyu Cai, Chuiqin Fan, Lichun Xie, Huifeng Zhong, Aijia Li, Siyu Lv, Maochuan Liao, Xixi Yang, Xing Su, Yue Wang, Hongwu Wang, Manna Wang, Peng Huang, Yulin Liu, Yu Wang, Yufeng Liu, Tianyou Wang, Yong Zhong, Lian Ma

**Affiliations:** 1 Department of Hematology and Oncology, Shenzhen Children’s Hospital of China Medical University, Shenzhen, 518038 China; 2 Department of Pediatrics, The Second Affiliated Hospital of Shantou University Medical College, Shantou, 515041 China; 3 Department of Pediatrics, The Third Affiliated Hospital of Guangzhou Medical University, The Women and Children’s Medical Center of Guangzhou Medical University, Guangzhou, 510150 China; 4 Department of Pediatrics, The First Affiliated Hospital of Zhengzhou University, Zhengzhou, 450052 China; 5 Department of Hematology and Oncology, Beijing Children’s Hospital, Capital Medical University, Beijing, 100045 China; 6 Department of Paediatrics, The Southeast General Hospital of Dongguan, Dongguan, 523000 China; 7 Shenzhen Public Service Platform of Molecular Medicine in Pediatric Hematology and Oncology, Shenzhen, 518000 China

**Keywords:** Mesenchymal stromal cells, Single-cell RNA sequencing, Heterogeneity of immunomodulatory function, Foreskin mesenchymal stromal cells, Human umbilical cord mesenchymal stromal cells

## Abstract

**Background:**

Mesenchymal stromal cells (MSCs) are heterogeneous populations. Heterogeneity exists within the same tissue and between different tissues. Some studies have found enormous heterogeneity in immunomodulatory function among MSCs derived from different tissues. Moreover, the underlying mechanism of heterogeneity in immunomodulatory abilities is still unclear.

**Methods:**

Foreskin mesenchymal stromal cells (FSMSCs) and human umbilical cord mesenchymal stromal cells (HuMSCs) were isolated and cultured until the third passage. According to the International Association for Cell Therapy standard, we confirmed the cell type. Then, FSMSCs and HuMSCs were cocultured with human peripheral blood mononuclear cells (PBMCs) stimulated by lipopolysaccharide (LPS) in vitro. Furthermore, the supernatant was sampled for an enzyme-linked immunosorbent assay to investigate the secretion of IL-1β, IL-6, IL-10, TNF-α, and TGF-β1. Finally, we performed single-cell RNA sequencing (scRNA-seq) of FSMSCs and HuMSCs.

**Results:**

We successfully identified FSMSCs and HuMSCs as MSCs. When cocultured with LPS pretreated PBMCs, FSMSCs and HuMSCs could effectively reduced the secretion of IL-1β and TNF-α. However, FSMSCs stimulated the PBMCs to secrete more IL-10, TGF-β1, and IL-6. Furthermore, 4 cell subsets were identified from integrated scRNA-seq data, including proliferative MSCs (*MKI67*^+^, *CD146*^*low*+^, *NG2*^+^, *PDGFRB*^*−*^), pericytes (*CD146*^*high*+^, *PDGFRB*^+^, *MKI67*^*−*^, *CD31*^*−*^, *CD45*^*−*^, *CD34*^*−*^), immune MSCs (*CXCL12*^*high*+^, *PTGIS*^*high*+^, *PDGFRB*^+^, *CD146*^*−*^, *MKI67*^*−*^) and progenitor proliferative MSCs (*CXCL12*^*low*+^, *PTGIS*^*low*+^, *PDGFRB*^+^, *CD146*^*−*^, *MKI67*^*−*^). Among them, we found that immune MSCs with strengthened transcriptional activity were similar to pericytes with regard to the degree of differentiated. Various of immune-related genes, gene sets, and regulons were also enriched in immune MSCs. Moreover, immune MSCs were determined to be close to other cell subsets in cell–cell communication analysis. Finally, we found that the proportion of immune MSCs in foreskin tissue was highest when comparing the subset compositions of MSCs derived from different tissues.

**Conclusions:**

FSMSCs show better immunomodulatory capacity than HuMSCs in vitro. Moreover, immune MSCs may play a vital role in the heterogeneity of immunoregulatory properties. This study provides new insights suggesting that immune MSCs can be isolated to exert stable immunoregulatory functions without being limited by the heterogeneity of MSCs derived from different tissues.

**Supplementary Information:**

The online version contains supplementary material available at 10.1186/s13578-022-00848-w.

## Introduction

Mesenchymal stromal cells (MSCs) are stromal cells that exist in a variety of tissues with multidirectional differentiation potential. They can differentiate into different cells, including osteoblasts, adipocytes, and chondrocytes. Moreover, MSCs can express the surface markers CD73, CD90 and CD105, but do not express HLA-DR, CD11b, CD19, CD34, and CD45 [1]. Furthermore, in addition to exhibiting potential for tissue differentiation, MSCs with powerful immunoregulatory abilities can also be applied for the treatment of immune-related diseases, such as graft-versus-host disease (GVHD) [2], multiple sclerosis [3], sepsis [4], systemic lupus erythematosus [5], Crohn’s disease [6], osteoarthritis [7] and acute lung injury with COVID-19 infection [8].

MSCs were first isolated from bone marrow. However, ethical disputes, the invasive nature of the operation, and low yield of in vitro culture has limited the clinical application of MSCs derived from bone marrow. Therefore, a new source of MSCs without the above deficiencies is urgently needed. MSCs have been successfully obtained from other tissues, such as adipose tissue [9], foreskin tissue [10], umbilical cord [11], and synovial fluid [12]. Foreskin tissue and umbilical cord tissue are considered good substitutes for bone marrow due to the less-invasive nature of collection, high clone proliferation potential in vitro, and their tremendous immunomodulatory abilities [10, 13]. They are considered biological wastes without ethical issues and are easy to collect from circumcision or natural birth; the operations are less invasive than bone marrow operations and do not cause other dangers to the donors. However, MSCs are heterogeneous populations. Heterogeneity exists within the same tissue and between different tissues. Some studies have found enormous heterogeneity in immunomodulatory function among MSCs derived from different tissues, such as bone marrow, adipose tissue, and umbilical cord tissue [13]. However, a comparison of the immunomodulatory properties of MSCs derived from foreskin tissue and the umbilical cord tissue has not been reported. Thus, it is necessary to compare their immunomodulatory abilities to contributes to their further application.

MSCs have already been demonstrated to exert immunomodulatory capabilities by direct cell–cell contact or the secretion of cytokines [14]. Nevertheless, the mechanism of the difference of immunomodulatory functions of MSCs derived from different tissues is still unclear. Some scholars believe the difference might be related to the heterogeneity of MSCs. For example, *NES*^+^ MSCs can significantly reduce macrophage infiltration and induce macrophage conversion to anti-inflammatory M2 phenotype [15]; *CD271*^+^ MSCs can significantly inhibit T lymphocyte proliferation [16] and have more substantial chondrogenic potential than other MSCs [17]; *CD106*^+^ MSCs can more effectively regulate helper T cells and secrete more cytokines than other MSCs [18]; *CD146*^+^ MSCs can inhibit the activation of Th17 cells [19] as well as promote the conversion of M2 type macrophages and improve inflammation or fibrosis of the knee [20]. These findings indicate that MSCs are a mixed-cell population that consists of different cell subsets with different biological functions.

Gene markers can help us to distinguish these cell subsets. However, a single gene marker may not fully define the biological function of an MSC subsets. Thus, we need to integrate multiple gene markers to distinguish cell subsets with different biological functions among MSCs. Single-cell RNA sequencing (scRNA-seq), an emerging technique, can detect gene expression differences among cells [21]. Furthermore, some studies have compared human umbilical cord mesenchymal stromal cells (HuMSCs) with MSCs derived from bone marrow [22], adipose tissue [23], and synovial fluid [12] through scRNA-seq and the results have reflected the heterogeneity of cell subsets of MSCs cultured in vitro. Therefore, scRNA-seq can be used to explore target MSC subsets in order to explain the differences in immunomodulatory abilities of MSCs derived from different tissues. Moreover, a comparison of scRNA-seq data between foreskin mesenchymal stromal cells (FSMSCs) and HuMSCs has not been reported. Therefore, we compared the immunomodulatory properties of FSMSCs and HuMSCs in vitro and performed scRNA-seq to investigate the potential mechanism of the difference in immunomodulatory function in this study.

## Methods

### Ethical approval

The Second Affiliated Hospital of Shantou University Medical College of China approved the study (institutional review board nos. 2020-11 and 2021-89). After cesarean sections or natural births, umbilical cords were collected with the informed consent of healthy donors (Second Affiliated Hospital of Shantou University Medical College of China, institutional review board no. 2021-89) ranging in age from 23 to 40 years. Moreover, we collected foreskin tissues after circumcision surgery from healthy donors with the informed consent of the donors’ parents (Shenzhen Children’s Hospital of China, institutional review board no. 2020-11) ranging in age from 4 to 15 years. Moreover, whole peripheral blood was collected with the informed consent of healthy donors (Shenzhen Children's Hospital of China, institutional review board no. 2020-11) ranging in age from 20 to 30 years. In all, we collected four umbilical cord samples, four foreskin tissue samples, and one whole peripheral blood sample. All tissues or cells were used only for research.

### Isolation, culture and expansion of MSCs

We collected the foreskin tissues and umbilical cords in sterile tubes containing sterile phosphate buffer solution (PBS). Under sterile conditions, the foreskin tissues and umbilical cords were cleaned with iodophor and PBS for 5 min in turn and then transferred to a 10-cm petri dish. Next, we separated dermis tissue from foreskin tissue, cut the foreskin tissue into pieces, and transferred the foreskin tissue to a 10-cm petri dish. We also separated the amniotic membrane, artery, and vein in the umbilical cord tissue. Then, we collected the white connective tissue between the amniotic membrane and blood vessels in a 10-cm petri dish. Afterward, the minced dermis tissue and white connective tissue were cultured in an incubator (37 °C, 5% CO_2_) with 5 mL of Dulbecco’s modified Eagle’s medium/nutrient mixture F-12 (DMEM/F12) (Gibco, USA) containing 10% fetal bovine serum (Gibco, USA) until primary cells migrated from the tissue fragments and attached to the plate. We trypsinized the cells with trypsin (BI, Israel) (the trypsin concentration was 0.25%) and passaged them when the cells became 80% confluent. Finally, the cells of the third passage were applied in the experiment.

### Surface markers of MSCs

According to the identification standard for MSCs of the International Association for Cell Therapy (ISCT) [1], we detected the expression of CD73, CD90, CD105, CD45, CD34, CD11b, CD19, and HLA-DR in third passage HuMSCs and FSMSCs by flow cytometry. First, we discarded the medium, washed the cells twice with PBS, and then used trypsin to digest the cells. Next, we stopped the digestion and adjusted the cell concentration to 1 × 10^6^ cells/mL. We prepared eight tubes and added 1 mL of suspended cells to each tube. Afterward, we added 5 µL of different antibodies, such as mouse anti-human FITC-CD90 (BioLegend, USA, catalog number: 328108, clone number: 5E10), mouse anti-human PerCP-Cy^TM^5.5-CD105 (BD, Germany, catalog number: 560819, clone number: 266), mouse anti-human APC-CD73 (BD, Germany, catalog number: 560847, clone number: AD2), mouse anti-human PE-HLA-DR (BD, Germany, catalog number: 555812, clone number: G46-6), mouse anti-human PE-CD11b (BioLegend, USA, catalog number: 301306, clone number: ICRF44), mouse anti-human PE-CD19 (BioLegend, USA, catalog number: 302208, clone number: HIB19), mouse anti-human PE-CD45 (BioLegend, USA, catalog number: 304008, clone number: HI30) and mouse anti-human PE-CD34 (BioLegend, USA, catalog number: 343606, clone number: 561), in different tubes and incubated the mixtures at 37 °C for 30 min in the dark. After incubation, we added 1 mL of PBS to wash out the excess antibodies and then centrifuged the cells at 250×*g* for 10 min. Finally, the cells were resuspended in 0.5 mL of PBS and analyzed in a FACSCanto II cytometer (BD Bioscience). Each tube recorded 20,000 cells. In addition, we prepared one tube with 1 mL of suspended cells without adding any antibody as a negative control. We processed the tube with the same process and recorded 20,000 cells to determine background fluorescence.

### Multidirectional differentiation

According to the ISCT identification standard for MSCs [1], we tested the osteogenic, adipogenic, and chondrogenic differentiation abilities of FSMSCs and HuMSCs. For osteogenesis, 6 × 10^4^ cells with 0.5 mL of MSC NutriStem® XF Medium (BI, 05-200-1) were seeded in each well of 24-well plates precoated with MSC Attachment Solution (BI, 05-752-1). The cells were cultured in an incubator (37 °C, 5% CO_2_) for 24–48 h to achieve > 80% confluence before induction in 0.5 mL of MSCgo™ osteogenic differentiation medium (BI, 05-440-1). Afterward, we incubated the cells for 10–21 days and changed the medium every 2–3 days. Finally, we used a 2% Alizarin Red S solution (VivaCell, 0195019) to evaluate osteogenesis. For adipogenesis, the cells were finally grown and induced in 0.5 mL MSCgo™ adipogenic complete medium that consisted of supplement mix I (BI, 05-331-1-01), supplement mix II (BI, 05-332-1-15), and MSCgo™ Adipogenic basal medium (BI, 05–330-1B). Next, we incubated the cells for 6–8 days and changed the medium every 2–3 days. After the cells became rounded and started floating, we replaced the MSCgo™ Adipogenic complete medium with MSC NutriStem® XF (BI, 05-200-1) and cultured the cells for 3–6 days. Once lipid droplets formed, Oil Red-O staining (VivaCell, 0175019) was used to evaluate adipogenesis. For chondrogenesis, 1 × 10^5^ cells with 0.1 mL of MSC NutriStem® XF Medium (BI, 05-200-1) were seeded in a 96-well plate. Then, the cells were cultured in an incubator (37 °C, 5% CO_2_) for 24 h to obtain spheroids. The culture medium was replaced with MSCgo™ Chondrogenic Differentiation Medium (BI, 05-220-1B). The cells were cultured for 14–21 days in an incubator (37 °C, 5% CO_2_) and the medium was changed every 3–4 days. Finally, Alcian Blue (VivaCell, 0185019) was used to evaluate chondrogenesis.

### Comparison of immunomodulatory function

HuMSCs and FSMSCs in the logarithmic growth phase were digested and then seeded in 12-well plates (1 × 10^6^ cells per well). The cells were cultured at 37 °C until the confluence reached 50%. Then, we discarded the supernatant and replaced it with 1 mL of fresh serum-free medium. Peripheral blood mononuclear cells (PBMCs) were isolated from whole peripheral blood by Ficoll-Hypaque gradient centrifugation. Then, we resuspended the PBMCs to 1 × 10^6^ cells/mL in serum-free medium, and the PBMCs were mixed with MSCs at a 10:1 ratio during the coculture. The experimental cells were divided into 4 groups: (a) PBMCs/HuMSCs/FSMSCs cultured alone, (b) PBMCs/HuMSCs/FSMSCs stimulated by lipopolysaccharide (LPS), (c) HuMSCs/FSMSCs cocultured with PBMCs without stimulation, and (d) HuMSCs/FSMSCs cocultured with LPS pretreated PBMCs. Furthermore, the final concentration of LPS (Sigma, USA) was 10 ng/mL. The cells were incubated for 24 h (37 °C, 5% CO_2_). Then, the cocultured supernatant was collected from different wells at 2 h, 4 h, 12 h and 24 h and centrifuged (1000×*g*, 5 min) to remove the cells. Finally, we detected the levels of secreted IL-1β, IL-6, IL-10, TNF-α, and TGF-β1 in the supernatant according to the manual of the enzyme-linked immunosorbent assay (ELISA) kit (Human IL-1β Elisa Kit: NeoBiosience, China, EHC002b.96; Human IL-6 Elisa Kit: NeoBiosience, China, EHC007.96; Human IL-10 Elisa Kit: NeoBiosience, China, EHC009.96; Human TNF-α Elisa Kit: NeoBiosience, China, EHC107b.96; Human TGF-β1 Elisa Kit: NeoBiosience, China, EHC107b.96). Three replicates were detected for each time point in the experimental group. The results are shown as the means ± standard error and visualized with a line graph. The Kruskal–Wallis test was used for comparisons among multiple independent samples, and Dunn’s test was used for pairwise comparison. A difference was considered statistically significant if the p value of the Kruskal–Wallis test or Dunn’s test was less than or equal to 0.05.

### Single-cell transcriptome sequencing

First, we digested third-passage of FSMSCs and HuMSCs with cell survival rate higher than 80%. Second, the concentration of cell suspension was adjusted to 1 × 10^6^ cells/mL for the 10× Genomics Chromium platform (Single Cell 3′ library and Gel Bead Kit V3). The cell sequencing counts of FSMSCs and HuMSCs were 15,950 and 15,972, respectively. Then, the samples were processed through following steps: GEM creation, thermal cycling, post cycling cleanup, cDNA amplification, library preparation, library quantification, and sequencing on an Illumina Hiseq3000. Finally, the sequencing results was mapped to the GRCh38 human genome, and the reads were counted by Cell Ranger software (version 3.1.0) [24].

### Data processing

We performed cell quality control and cluster analysis for the scRNA-seq data with the R package Seurat (version 4.0.0) [25]. The R version was 4.0.4. First, we used the Read10X function of the R package Seurat (version 4.0.0) [25] to load the count matrix and create the Seurat object. Second, we filtered the low-quality cells with fewer than 200 genes, more than 20,000 genes, more than 10% mitochondrial genes, and more than 10% erythrocyte genes. Then, we ran normalization to correct the depth of cells with the NormalizeData function (LogNormalize) and removed the effect of the mitochondrial genes during scaling. We also executed principal component analysis (PCA) through the first 2000 genes with high heterogeneity and evaluated the first 50 principal components (PCs) with JackStraw and ElbowPlot functions of the R package Seurat (version 4.0.0) [25]. We calculated the percentages of each principal component in the population variance. Moreover, the first 20 PCs with the most significant p values were used for uniform manifold approximation and projection (UMAP). We also used the first 20 PCs to cluster cells, and the clustering resolution was 0.5. Finally, we annotated the clusters based on the surface markers of MSCs [1].

### Integration analysis

First, we integrated the cell subsets annotated as MSCs among FSMSCs and HuMSCs with the FindIntegrationAnchors and IntegrateData function of the R package Seurat (version 4.0.0) [25]. We used canonical correlation analysis to remove the batch effect. Then, we assessed the correction of batch effects based on PCA plots before and after integration. The integrated data were normalized, reduced, and clustered again. Furthermore, the first 20 PCs with the most significant p values were used for t-distributed stochastic neighbor embedding (TSNE). In addition, we used the CellCycleScoring function of the R package Seurat (version 4.0.0) [25] to infer the cell cycle phase of each subset. Moreover, we constructed a clustering tree based on the similarity of cell subsets with the BuildClusterTree function of the R package Seurat (version 4.0.0) [25] and visualized it with a dendrogram using the R package ggtree (version 2.4.1) [26]. Finally, we annotated these clusters according to the literature [1, 27–31].

### Trajectory inference

Trajectory inference, also known as pseudotime analysis, can sort individual cells in pseudotime and simulate dynamic changes during development based on the expression patterns of essential genes. We used the R package slingshot (version 1.8.0) [32] to infer the possible differentiation trajectories. We also used the R package CytoTRACE (version 0.3.3) [33] to infer the differentiatION degreeS of MSC subsets in order to determine the possible starting points of the trajectory.

### RNA velocity analysis

RNA velocity analysis can identify the transcriptional statuses of different cell subsets based on changes in mRNA abundance. First, we used the Python package velocyto.py (version 0.17.17) [34] to generate the loom file containing spliced, unspliced, and ambiguous mRNA. Second, we evaluated the transcriptional statuses of different subsets by distinguishing unspliced mRNA from spliced mRNA with the help of the R package velocyto.R (version 0.6.0) [34].

### Gene set enrichment analysis

Gene Set Enrichment Analysis (GSEA) can be used to infer whether a predefined gene set is enriched with a specific cell type to reflect its biological functions. First, we used the R package msigdbr (version 7.2.1) [35] to download the hallmark gene sets. Next, we calculate the enrichment scores and p values of hallmark gene set in different subpopulations of MSCs with the R package singleseqgset (version 0.1.2.9000) [36], through which we were able to assess the potential biological function for every subset.

### Immune-related differentially expressed genes

We compared the gene expression between FSMSCs and HuMSCs with the Findallmarker function of the R package Seurat (version 4.0.0) [25] and filtered the differentially expressed genes with corrected p values ≤ 0.05 and absolute log_2_ fold change (log_2_FC) values ≥ 2. We also calculated the marker genes in different MSC subsets with the Findallmarker function of the R package Seurat (version 4.0.0) [25] with a corrected p value ≤ 0.05 and log_2_FC ≥ 0.8.

To analyze potential immune functions, we extracted 2073 immune-related genes (Additional file 5) from the ImmPort database (https://www.immport.org/shared/genelists, access time: July 7th, 2020) [37] and MSigDB database (Gene Ontology database, release version: 7.2) [38]. Then, we intersected these immune-related genes with the differentially expressed genes and divided these intersected genes into different immune categories according to the related literature, the ImmPort database, and the GeneCards database [39]. Finally, we visualized the expression of intersecting genes with the R packages ComplexHeatmap (version 2.6.2) [40] and circlize (version 0.4. 12) [41].

### Gene regulatory networks analysis

pySCENIC (version 0.10.3) enabled us to infer single-cell gene regulatory networks from the scRNA-seq data [42]. The Python version was 3.6.12. Briefly, the coexpression modules containing different transcription factors and their target genes were first inferred from the scRNA-seq matrix. Then, the significantly enriched motifs were retained in the coexpression module by motif enrichment analysis, and the target genes of the module that were less correlated with the significant motif were removed. The coexpression module's transcription factor and remaining target genes were regarded as the regulon. Next, the regulon activity scores (RASs) of the different regulons in each cell were assessed, and the RAS threshold per regulon was calculated. The regulon was considered activated in the cell when the RAS was greater than the threshold, and vice versa. We performed the “0/1” conversion for the RAS matrix based on the threshold and created a binary matrix to best show the differences in the regulon in various cell types and eliminate technical bias. Finally, we calculated the regulon-specific scores (RSSs) in different MSC subsets by using the R package philentropy (version 0.4.0) [43]. The regulons with RSSs > 0.6 were considered cell-type-specific regulons (CTSRs).

### Cell–cell communication analysis

CellChat (version 1.0.0) was used to infer and visualize intercellular communication networks from the scRNA-seq data [44]. First, the CellChat object was created, and the database was set to the human ligand–receptor interaction database. Next, the overexpressed ligands or receptors were identified, and the gene expression data were projected onto the protein–protein interaction network. A probability value was then assigned to each ligand–receptor interaction, and permutations were performed to infer biologically meaningful interactions. Then, the probabilities of all interactions associated with each signaling pathway were summarized, and the probability of communication for each signaling pathway was calculated. The roles of each cell subset were identified at the signaling pathway level by calculating the network centrality. Finally, several signaling groups were identified by quantifying the functional similarity of all significant signaling pathways.

### Comparison of MSCs isolated from different tissue sources

To evaluate the ideal tissue source for isolating immune MSCs, we downloaded scRNA-seq data from the NCBI public database for adipose mesenchymal stromal cells (ADMSCs) (SRP148833) [9] and bone marrow mesenchymal stromal cells (BMSCs) (GSE115149, GSE162692) [45, 46]. The SRP148833 database was processed by Cell Ranger software (version 3.1.0) [24], with which we were able to acquire the gene expression matrix of the ADMSCs. For the GSE115149 and GSE162692 datasets, only the gene expression matrices of untreated BMSCs were retained. After obtaining the gene expression matrices, the data were processed according to the same quality control process as described above. Then, we evaluated the proportions of the subsets of ADMSCs and BMSCs based on the integrated data above with the R package SingleR (version 1.4.0) [47]. Finally, the proportions of the subsets of MSCs derived from different tissues were visualized with a circle plot.

## Results

### Identification of FSMSCs and HuMSCs

**Fig. 1:**
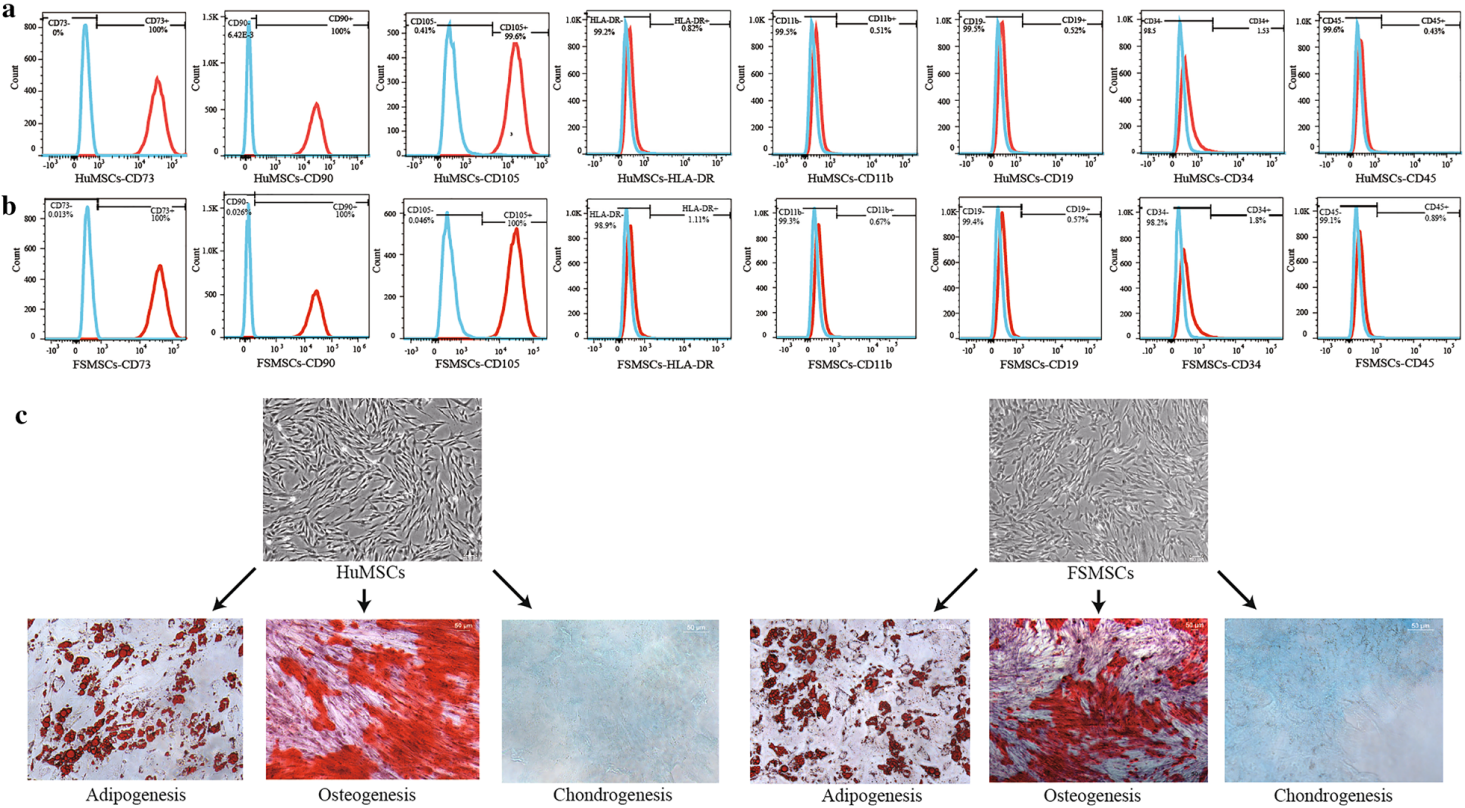
Identification of FSMSCs and HuMSCs. **a** Flow cytometric analysis of surface markers in HuMSCs. The control cells without staining are blue, while the positive cells with staining are red. **b** Flow cytometric analysis of surface markers in FSMSCs. The control cells without staining are blue, while the positive cells with staining are red. **c** Adipogenic, osteogenic, and chondrogenic differentiation of HuMSCs and FSMSCs

According to the minimum identification standard about MSCs of the ISCT, the cells isolated from foreskin tissue and umbilical cord were confirmed as MSCs, respectively. First, FSMSCs and HuMSCs were able to attach plastic surfaces, and they exhibited a typical long, spindle-shaped fibroblast-like morphology under the microscope. Second, positive surface markers, such as CD73, CD90, and CD105, were expressed in more than 95% of the cells, while negative surface markers, such as CD45, CD34, CD11b, CD19, and HLA-DR, were expressed in fewer than 2% of the FSMSCs and HuMSCs (Fig. 1a, b). Red spherical vacuoles were observed by Oil Red O staining after induction of adipogenic differentiation in FSMSCs or HuMSCs (Fig. 1c). This finding indicated that lipid droplets formed in these cells. After the induction of osteogenic differentiation, scattered round crimson nodules were observed in FSMSCs or HuMSCs by 2% ARS staining (Fig. 1c). Massive amounts of calcium were deposited in the cytoplasm. Finally, blue acid mucopolysaccharides were visible in FSMSCs or HuMSCs by Alcian Blue staining after the induction of chondrocyte differentiation (Fig. 1c).

### Immunomodulatory abilities of FSMSCs and HuMSCs

**Fig. 2:**
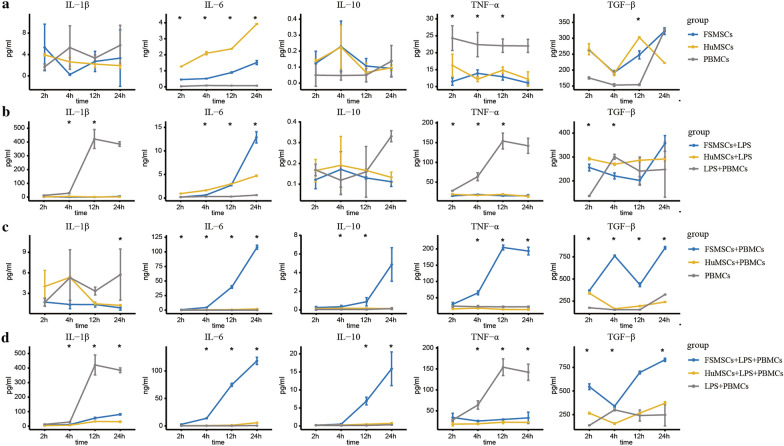
Results of ELISAs. **a** Secretion of inflammation-related cytokines in different groups. The blue line represents FSMSCs, the yellow line represents HuMSCs, and the gray line represents PBMCs. Moreover, an asterisk indicates that the p value of the Kruskal–Wallis rank sum test among different groups at the specified time point was less than 0.05. **b** Secretion of inflammation-related cytokines in different groups. The blue line represents FSMSCs stimulated by LPS, the yellow line represents HuMSCs stimulated by LPS, and the gray line represents PBMCs stimulated by LPS. Furthermore, an asterisk indicates that the p value of the Kruskal–Wallis rank sum test among different groups at the specified time point was less than 0.05. **c** Secretion of inflammation-related cytokines in different groups. The blue line represents FSMSCs cultured with PBMCs, the yellow line represents HuMSCs cultured with PBMCs, and the gray line represents PBMCs cultured alone. An asterisk indicates that the p value of the Kruskal–Wallis rank sum test among different groups at the specified time point was less than 0.05. **d** Secretion of inflammation-related cytokines in different groups. The blue line represents FSMSCs cultured with PBMCs stimulated by LPS, the yellow line represents HuMSCs cultured with PBMCs stimulated by LPS, and the gray line represents PBMCs stimulated by LPS. An asterisk indicates that the p value of the Kruskal–Wallis rank sum test among different groups at the specified time point was less than 0.05

The secretion of IL-1β, IL-6, IL-10, TNF-α, and TGF-β1 was measured to compare the immunomodulatory abilities of FSMSCs and HuMSCs through ELISA. First, we analyzed the differences in autocrine inflammation-related cytokines among FSMSCs, HuMSCs, and PBMCs (Fig. 2a, Additional file 1: Tables S1–S4). The differences in the basal secretion of IL-1β, IL-10, and TGF-β1 in these three cell types were not statistically significant at any time point. The basal secretion of IL-6 was significantly higher in HuMSCs than in FSMSCs and PBMCs, while there was no significant difference between FSMSCs and PBMCs. The basal secretion of TNF-α was significantly higher in PBMCs than in FSMSCs and HuMSCs, while there was no significant difference between FSMSCs and HuMSCs. In addition, we analyzed whether there were differences in the basal secretion of inflammation-related cytokines among these three cell types at different time points (Fig. 2a, Additional file 1: Table S1–S4). The basal secretion level of IL-6 increased with time, while the basal secretion level of TGF-β1 showed a fluctuating pattern. The differences in the secretion of IL-1β, IL-10, and TNF-α were not statistically significant at different time points.

Next, we analyzed the differences in the secretion of inflammation-associated cytokines among FSMSCs, HuMSCs, and PBMCs under LPS stimulation. The differences in the secretion of IL-10 and TGF-β1 were not statistically significant. The secretion of IL-1β and TNF-α was highest in PBMCs, while the secretion level of IL-6 was highest in FSMSCs. In addition, we analyzed whether there were differences in the secretion of inflammation-associated cytokines among MSCs at different time points after stimulation with LPS (Fig. 2b, Additional file 2: Table S5–S8). The secretion of IL-6 increased with time, while the differences in the secretion of IL-10 were not statistically significant. The secretion of IL-1β and TNF-α in PBMCs increased with time. The secretion of TGF-β1 in FSMSCs increased with time.

Moreover, we analyzed the differences in the secretion of inflammation-related cytokines among FSMSCs and HuMSCs cocultured with PBMCs and PBMCs cultured alone (Fig. 2c, Additional file 3: Table S9–S12). Overall, the secretion levels of IL-6, TNF-α, IL-10, and TGF-β1 were higher in FSMSCs cocultured with PBMCs than in HuMSCs cocultured with PBMCs or in PBMCs cultured alone. There was no significant difference in the secretion of IL-1β among these three conditions. In addition, we analyzed whether there were differences in the secretion of inflammation-related cytokines at different time points among these three conditions (Fig. 2c, Additional file 3: Table S9–S12). The secretion of IL-6 increased with time. The secretion levels of IL-10 and TNF-α in FSMSCs cocultured with PBMCs increased with time.

Finally, we mimicked the inflammatory response by stimulating PBMCs with LPS in vitro. FSMSCs and HuMSCs were cocultured with PBMC prestimulation. Then, we compared the immunoregulatory abilities by detecting the secretion of inflammation-related cytokines (Fig. 2d, Additional file 4: Table S13–S16). The secretion of the proinflammatory cytokines IL-1β and TNF-α was lower in the FSMSCs and HuMSCs than in the control cells. The secretion of the bidirectional immunomodulatory cytokine IL-6 and the anti-inflammatory cytokines IL-10 and TGF-β1 was higher in FSMSCs than in HuMSCs. In addition, we investigated whether there were significant differences in the secretion levels of inflammation-associated cytokines in the FSMSCs and HuMSCs at different time points (Fig. 2d, Additional file 4: Table S13–S16). IL-6, IL-10, and TGF-β1 secretion increased with time in FSMSCs cocultured with prestimulated PBMCs. IL-10 secretion increased with time in HuMSCs cocultured with prestimulated PBMCs. In summary, FSMSCs and HuMSCs both inhibited the secretion of the proinflammatory cytokines IL-1β and TNF-α from PBMCs. FSMSCs were more significantly able to upregulate the secretion of the bidirectional immunomodulatory cytokine IL-6 and the anti-inflammatory cytokines IL-10 and TGF-β1 than HuMSCs.

### Quality control, clustering and biological annotation

**Fig. 3:**
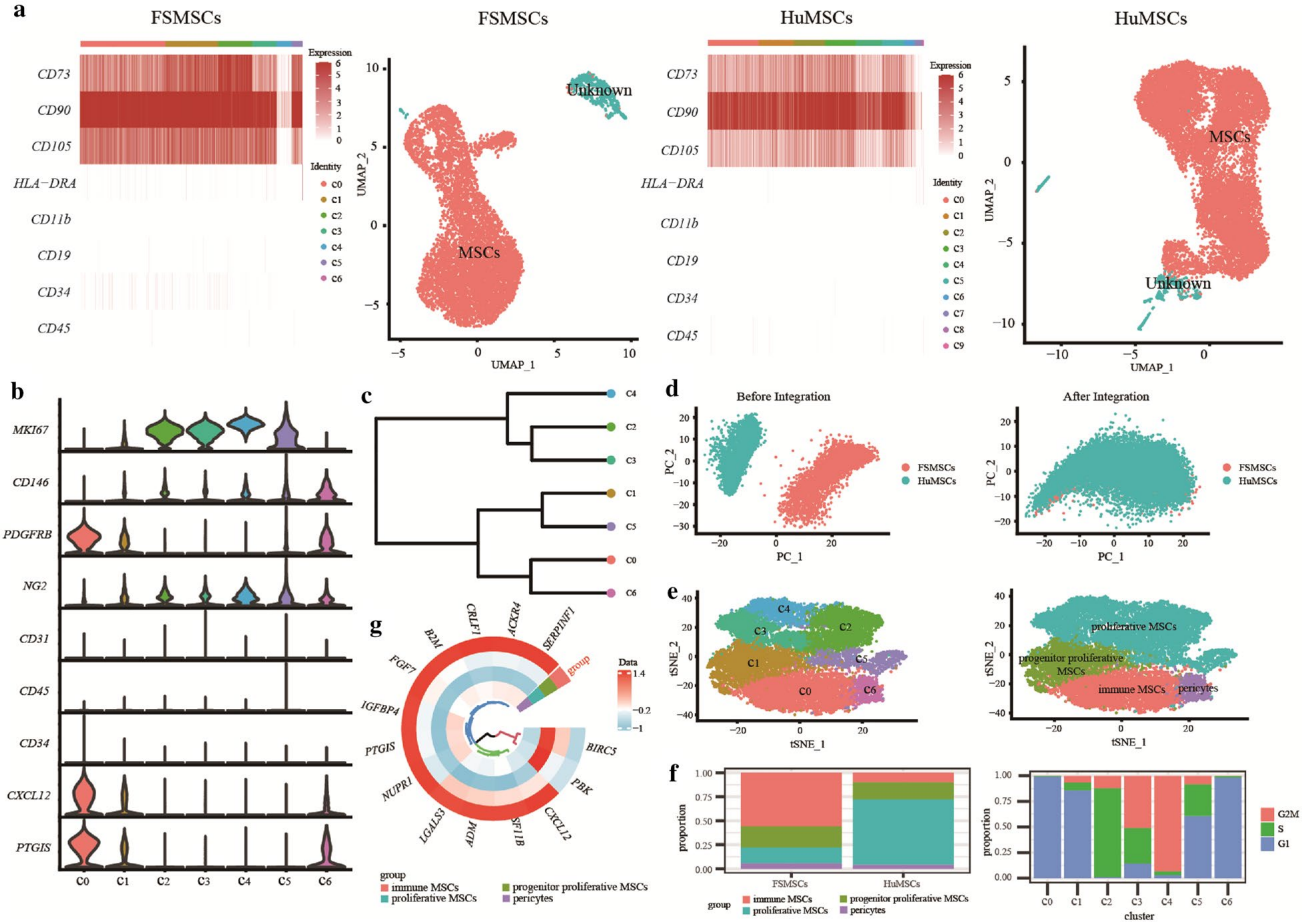
Visualization of bioinformatics analysis results. **a** Heatmap plots showing the expression of MSC surface markers. The bar chart above each heatmap represents the different cell subsets with different colors. Moreover, the UMAP plots show the distributions of different cell subsets in low-dimensional space. Red dots represent MSCs, and green dots represent unknown cells. **b** Violin plots showing the distribution of the expression of marker genes among different MSC subsets with different colors. **c** Dendrogram plot showing the similarity of MSC subsets. Dots with different colors correspond to different MSC subsets. The expression patterns of MSC subsets are closer when the dots are closer together in the dendrogram plot. **d** PCA plots showing the distribution of FSMSCs and HuMSCs in low-dimensional space before and after integration. Red dots represent FSMSCs, and green dots represent HuMSCs. The left plot shows the batch effect of the scRNA-seq data before integration. The right plot shows the batch effect of the scRNA-seq data after integration. **e** TSNE plots showing the distribution of different MSC subsets in low-dimensional space before and after annotation. **f** The left bar plots show the proportions of different MSC subsets between FSMSCs and HuMSCs. Different colors represent different MSCs subsets. The right bar plots show the proportions of different cell cycle phases among different MSC subsets. Different colors represent different cell cycle phases. **g** Circle plot showing the expression of the immune-related differentially expressed genes in immune MSCs. The color intensity of the grid represents the degree of gene expression. A bluer color indicates lower gene expression

Quality control, clustering, and biological annotation were performed for FSMSCs and HuMSCs. First, the Seurat objects for FSMSCs and HuMSCs were created separately. Second, quality control was performed, and low-quality cells with fewer than 200 genes, more than 20,000 genes, more than 10% mitochondrial genes and more than 10% erythrocyte genes were filtered. Then, we obtained 7335 FSMSCs, in which the median UMI was 33202 and the median gene number was 5440. Moreover, we obtained 12542 HuMSCs, in which the median UMI was 15191 and the median gene number was 3831. This result indicated that the quality of the scRNA-seq data was high. Afterward, normalization was run, and the effect of the mitochondrial genes on scaling was removed. Next, we executed dimensionality reduction by PCA through the first 2000 genes with high heterogeneity and evaluated the first 50 PCs. We evaluated the first 50 PCs with the JackStraw and ElbowPlot functions of the R package Seurat (Additional file 11: Plot S1, Additional file 12: Plot S2). We also calculated the percentages of each principal component in the population variance (Additional file 10). We found that the first 20 PCs with standard deviations could explain most of the variability in the data (Additional file 11: Plot S1b, Additional file 12: Plot S2b). Thus, the first 20 PCs with the most significant p values (Additional file 11: Plot S1a, Additional file 12: Plot S2a) were used for UMAP. The first 20 PCs were also used to cluster cells with a resolution of 0.5, and we ultimately obtained seven clusters of FSMSCs and 10 clusters of HuMSCs (Fig. 3a).

Then, the clusters were annotated according to the surface markers of MSCs [1]. Among FSMSCs, those in C0, C1, C2, C3, and C5 highly expressed the positive marker genes *CD73*, *CD90* and *CD105* and lacked expression of the negative marker genes *HLA-DRA*, *CD11b*, *CD19*, *CD34*, and *CD45* (Fig. 3a). This finding indicated that the cell in these clusters were possible MSCs. However, C4 cells highly expressed HLA-DRA, and C6 cells lacked expression of *CD73* and *CD105*. This result indicated that C4 and C6 cells did not satisfy the identification criteria for MSCs. Similarly, among HuMSCs, the cells in C0, C1, C2, C3, C5, and C6 highly expressed the positive marker genes *CD73*, *CD90* and *CD105* and lacked expression of the negative marker genes *HLA-DRA*, *CD11b*, *CD19*, *CD34*, and *CD45*, suggesting that the cells in these clusters might be MSCs. Nevertheless, C4, C7, C8, and C9 cells lacked expression of *CD73* and *CD105*, indicating that the cells in these clusters did not satisfy the identification criteria for MSCs (Fig. 3a). In summary, the cells in C0, C1, C2, C3, and C5 were MSCs among FSMSCs and accounted for 93.05% of the FSMSCs. The cells in C0, C1, C2, C3, C5, and C6 were MSCs among HuMSCs and accounted for 95.85% of the HuMSCs.

### Integration analysis

We integrated the cell subsets annotated as MSCs among FSMSCs and HuMSCs to reveal the potential heterogeneity of MSCs (Fig. 3a). Then, the batch effect was removed by canonical correlation analysis and evaluated with a PCA plot. The FSMSCs and HuMSCs were able to overlap well after integration (Fig. 3d), indicating that the batch effect was corrected well. Afterward, the integrated data were normalized and scaled again. We also executed dimensionality reduction by PCA through the first 2000 genes with high heterogeneity and evaluated the first 50 PCs. We evaluated the first 50 PCs with the JackStraw and ElbowPlot functions of the R package Seurat (Additional file 13: Plot S3). We also calculated the percentages of each principal component in the population variance (Additional file 10). We found that the first 20 PCs with standard deviations could explain most of the variability in the data (Additional file 13: Plot S3b). Thus, the first 20 PCs with the most significant p values (Additional file 13: Plot S3a) were used for TSNE. The first 20 PCs were also used to cluster cells with a resolution of 0.5, through which seven major clusters were obtained (Fig. 3e). Furthermore, we calculated the proportions of these clusters between FSMSCs and HuMSCs, as shown on the bar plot (Fig. 3f). Moreover, the cell cycle phase of each cluster was inferred with the CellCycleScoring function of the R package Seurat (version 4.0.0) [25]. There were elevated proportions of G1 phase cells in C0, C2, and C6, suggesting a resting state. However, G2/M or S phase cells occupied large proportions of C2, C3, C4, and C5 cells (Fig. 3f). In addition, *MKI67*, a gene that encodes a nuclear protein associated with cell proliferation [27], was highly expressed in C2, C3, C4, and C5 cells (Fig. 3b). This result suggested that the proliferative activity of the cells in C2, C3, C4, and C5 was more substantial than that of the cells in the other subsets. Intriguingly, this result is consistent with the results of previous studies on MSCs cultured in vitro. Three morphologies of MSCs cultured in vitro have been reported: spindle-shaped, large and flattened, and small and prototypical. The first two kinds of cells are more extensive and proliferate more slowly. The latter cells, known as rapid self-renewing cells, [28, 29]. The results indicated that some cell subsets were more active in proliferation than others. Therefore, the cells in C2, C3, C4, and C5 were named proliferative MSCs (Fig. 3e).

Second, C6 cells highly expressed the positive marker genes *CD146*, *PDGFRB* and *NG2* and lacked expression of the negative marker genes *CD31*, *CD45* and *CD34* (Fig. 3b). These genes have been demonstrated to be markers of pericytes, but pericytes can also express the markers of MSCs [30]. Therefore, C6 cells were regarded as possible pericytes (Fig. 3e).

In addition, *CXCL12* and *PTGIS* were highly expressed in C0 cells, while *MKI67* and *CD146* lacked expression in C0 cells (Fig. 3b). Among these, *CXCL12* plays an essential role in the immunoregulatory function of MSCs [48]. *PTGIS*, prostacyclin I2 synthase, can catalyze the conversion of prostaglandin H2 to prostaglandin I2, which plays an essential role in immunoregulatory function [31]. We also observed that C1 and C6 cells expressed *CXCL12* and *PTGIS*, but the expression levels in these cells were much lower than those in C0 cells. This result suggested that C0, C1, and C6 cells all possessed immunomodulatory abilities. However, the C0 cells exhibited the strongest immunomodulatory function among these cells. C6 cells were identified as pericytes, which are known to exhibit immunomodulatory function [49]. Furthermore, C1 cells expressed low levels of *CXCL12*, and *PTGIS* and lacked *MKI67* and *CD146* expression (Fig. 3b). In addition, a few C1 cells expressed *MKI67* (Fig. 3b). Therefore, we constructed a clustering tree based on the similarities of MSC subsets and visualized it with a dendrogram plot. Then, we found that the expression patterns of C1 and C5 cells were closer than those of other cells (Fig. 3c). Moreover, the C5 cells belonged to the proliferative MSCs. The C1 cells were possible precursor cells of C5 cells, while the C1 cells might have represented a transitional stage from C0 cells to proliferative MSCs. Therefore, C1 cells were named progenitor proliferative MSCs, and C0 cells were named immune MSCs (Fig. 3e).

Finally, the proportion of subsets of FSMSCs and HuMSCs were calculated (Fig. 3f). Among them, proliferative MSCs made up 16% of FSMSCs and 68% of HuMSCs, pericytes made up 6% of FSMSCs and 4% of HuMSCs, immune MSCs made up 56% of FSMSCs and 10% of HuMSCs, and progenitor proliferative MSCs made up 22% of FSMSCs and 18% of HuMSCs.

### Trajectory inference

**Fig. 4:**
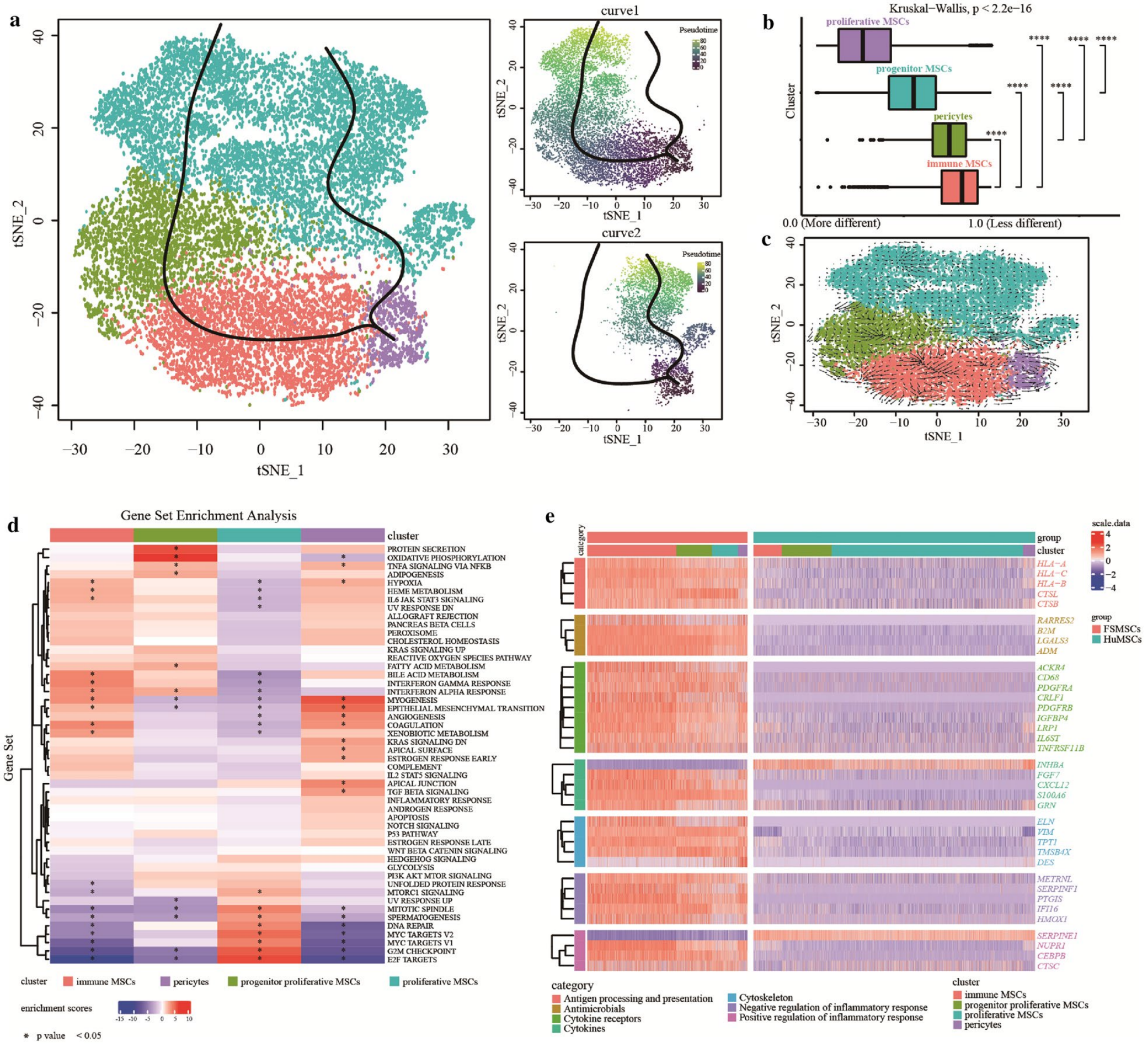
Results of trajectory inference, RNA velocity analysis, gene set enrichment analysis, and differential gene expression analysis. **a** TSNE plots showing the two differentiation trajectories. A larger pseudotime value is indicated by a greener dot color. **b** Box plot showing the distributions of CytoTRACE scores in different MSC subsets. A CytoTRACE score near 1.0 indicates a lower degree of differentiation, and vice versa. An asterisk indicates that the p value of the Wilcoxon test between the two groups was less than 0.0001. The p value of the Kruskal–Wallis test for all groups was less than 2.2e−16. **c** TSNE plot showing the distribution of transcriptional activity. A longer arrow indicates stronger transcriptional activity. **d** Heatmap showing the results of the hallmark gene set enrichment analysis. The rows are gene sets, and the columns are cell subsets. A bluer grid color indicates lower enrichment, and vice versa. The left dendrogram of the heatmap represents the similarity of expression patterns in the different gene sets. An asterisk indicates that the p value was less than 0.05. **e** Heatmap showing the expression of 37 immune-related differentially expressed genes between FSMSCs and HuMSCs. The rows are genes, and the columns are different groups or clusters. A bluer grid color indicates lower gene expression, and vice versa. The left dendrogram of the heatmap represents the similarity of expression patterns in the genes. Furthermore, the left bar with different colors represents different categories

The CytoTRACE package was used to infer the differentiation degrees of four MSC subsets (Fig. 4b). A CytoTRACE score nearer to 1.0 indicated a lower degree of differentiation, while a CytoTRACE score nearer to 0.0 indicated a higher degree of differentiation. The degrees of differentiation of proliferative MSCs and progenitor proliferative MSCs were higher than those of immune MSCs and pericytes (Fig. 4b). Some studies have reported that MSCs might originate from pericytes [50]. Therefore, we defined pericytes as the starting point of the differentiation trajectory of MSCs. Next, we constructed two differentiation trajectories based on transcriptional similarity with the R package slingshot (Fig. 4a). In the first trajectory, the cells started from pericytes and differentiated into proliferative MSCs. In the second trajectory, the cells started from pericytes, passed through the immune MSC and progenitor proliferative MSC stages, and ultimately differentiated into proliferative MSCs.

### RNA velocity analysis

The transcriptional activity of MSC subsets was inferred by RNA velocity analysis, in which a longer arrow indicates stronger transcriptional activity. We found that most of the arrows for immune MSCs were longer than those for proliferative MSCs (Fig. 4c). This finding indicated that the overall transcriptional activity of immune MSCs was stronger than that of proliferative MSCs.

### Gene set enrichment analysis

All cell subsets were subjected to gene set enrichment analysis to assess the potential biological functions of the subsets. First, the enrichment score and adjusted p value of the hallmark gene set of different MSC subsets were calculated (Additional files 6, 7) and then visualized with a heatmap plot (Fig. 4d). The asterisks on the heatmap plot indicate that the p value of the hallmark gene set was less than 0.05.

First, we found 15 statistically significant gene sets with adjusted p values ≤ 0.05 that were filtered from immune MSCs (Fig. 4d). Among them, the immune-related gene sets “IL6 JAK STAT3 SIGNALING”, “INTERFERON GAMMA RESPONSE” and “INTERFERON ALPHA RESPONSE” were upregulated in immune MSCs. The mitosis-related gene sets “MITOTIC SPINDLE”, “G2M CHECKPOINT” and “E2F TARGETS” were downregulated in immune MSCs. Second, 13 and 19 statistically significant gene sets were filtered by a corrected p value ≤ 0.05 from progenitor proliferative MSCs and pericytes, respectively. The immune-related gene set “TNFA SIGNALING VIA NFKB” was upregulated in both progenitor proliferative MSCs and pericytes. Furthermore, in progenitor proliferative MSCs, gene sets related to adipose differentiation, such as “ADIPOGENESIS” and “FATTY ACID METABOLISM”, were preferentially enriched. However, in pericytes, gene sets related to mesenchymal histogenesis, such as “MYOGENESIS”, “ANGIOGENESIS” and “EPITHELIAL MESENCHYMAL TRANSITION”, were preferentially enriched. Finally, 20 statistically significant gene sets with corrected p values ≤ 0.05 were filtered for proliferative MSCs. These cells lacked enrichment of the hypoxia-related gene set “HYPOXIA” and the immune-related gene sets “IL6 JAK STAT3 SIGNALING”, “INTERFERON GAMMA RESPONSE” and “INTERFERON ALPHA RESPONSE”. Nevertheless, the mitosis-related gene sets “MITOTIC SPINDLE”, “G2M CHECKPOINT” and “E2F TARGETS” were significantly upregulated in proliferative MSCs.

### Immune-related differential gene expression analysis

First, we compared the gene expression between FSMSCs and HuMSCs and filtered 205 differentially expressed genes with adjusted p values ≤ 0.05 and absolute log2FC values ≥ 2 (Additional file 8). Then, we intersected the immune-related genes with the 205 differentially expressed genes and finally obtained a total of 37 immune-related differentially expressed genes. We divided these 37 intersecting genes into seven immune categories: “Antigen processing and presentation”, “Antimicrobials”, “Cytokine receptors”, “Cytokines”, “Cytoskeleton”, “Negative regulation of inflammatory response” and “Positive regulation of inflammatory response”. Then, we visualized the expression of these 37 immune-related differentially expressed genes between FSMSCs and HuMSCs with a heatmap plot (Fig. 4e). Second, we compared the gene expression among different MSC subsets and filtered 84 differentially expressed genes in immune MSCs with adjusted p values ≤ 0.05 and absolute log2FC values ≥ 0.8 (Additional file 9). Then, we also intersected the immune-related genes with the 84 differentially expressed genes and finally obtained a total of 14 immune-related differentially expressed genes. We visualized the expression of these 14 intersecting genes among different MSC subsets (Fig. 3g).

### Gene regulatory networks analysis

**Fig. 5:**
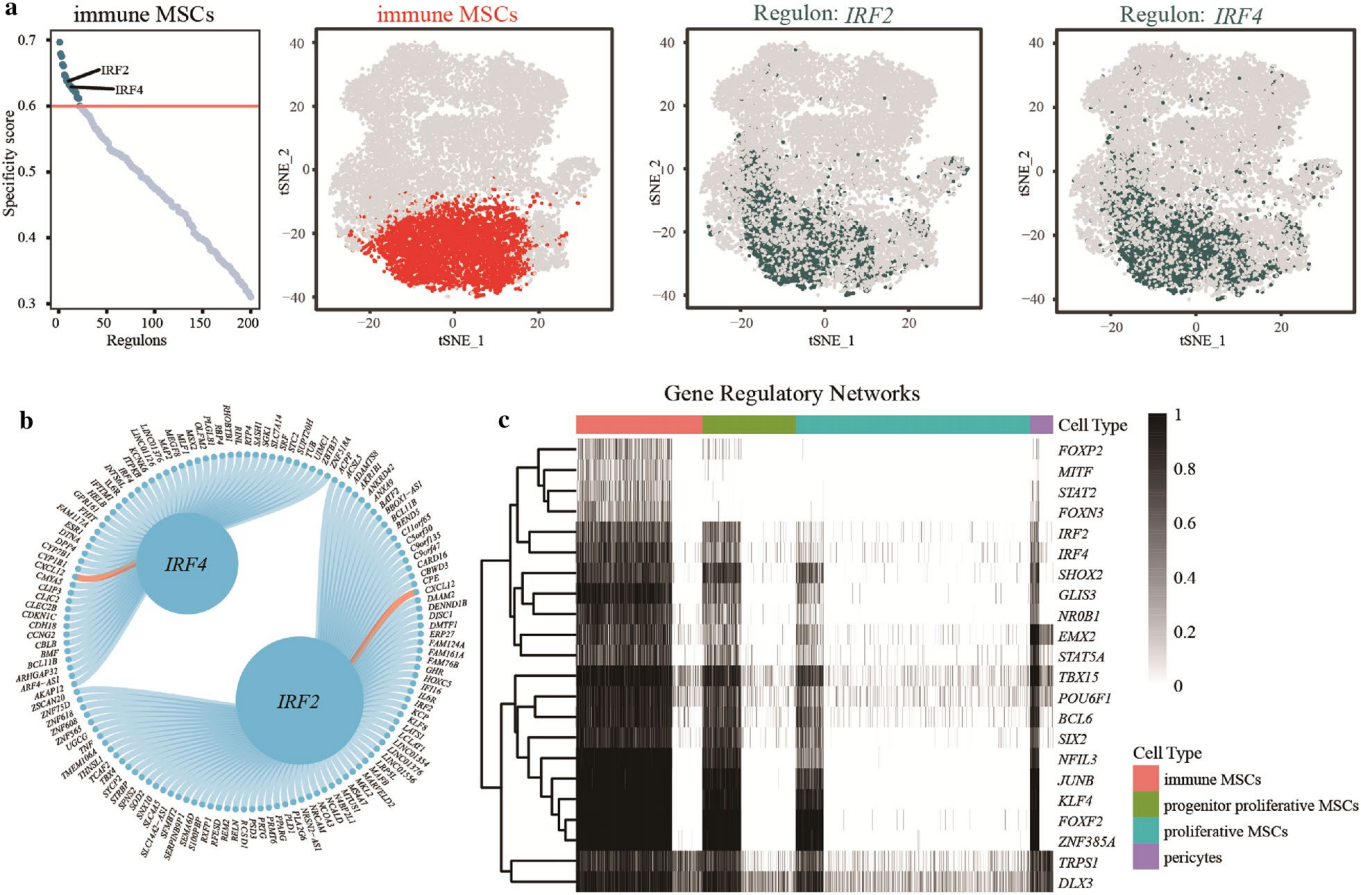
Results of gene regulatory network analysis. **a** The scatter plot shows the regulon-specific scores (RSSs) of all regulons and the threshold value of RSS with the red line. Only the regulons with RSSs > 0.6 were considered cell type-specific regulons (CTSRs). The *IRF2* and *IRF4* regulons are marked on the scatter plot. The TSNE plots show the distributions of MSC subsets and *IRF2* and *IRF4* regulon expression. **b** Circle plot showing the target genes of the *IRF2* and *IRF4* regulons. A higher number of target genes is indicated by a larger circle of the regulon. Each line represents a target gene, and the red line is CXCL12. **c** Heatmap plot showing the expression of 22 CTSRs among different cell subsets. A darker grid color indicates higher expression of the regulon. The left dendrogram of the heatmap represents the similarity of expression patterns in the regulon. The top bar of the heatmap represents different cell subsets

A gene regulatory network was constructed, and 22 cell type specific regulons (CTSRs) were identified in immune MSCs. The immune-related regulons *IRF2* and *IRF4* were highly expressed in immune MSCs (Fig. 5a). Interestingly, *IRF2* and *IRF4* might regulate the expression of target gene *CXCL12* based on the integrated data (Fig. 5b). Finally, we showed the expression of these 22 CTSRs among various MSC subsets with a heatmap plot (Fig. 5c).

### Cell–cell communication analysis

**Fig. 6:**
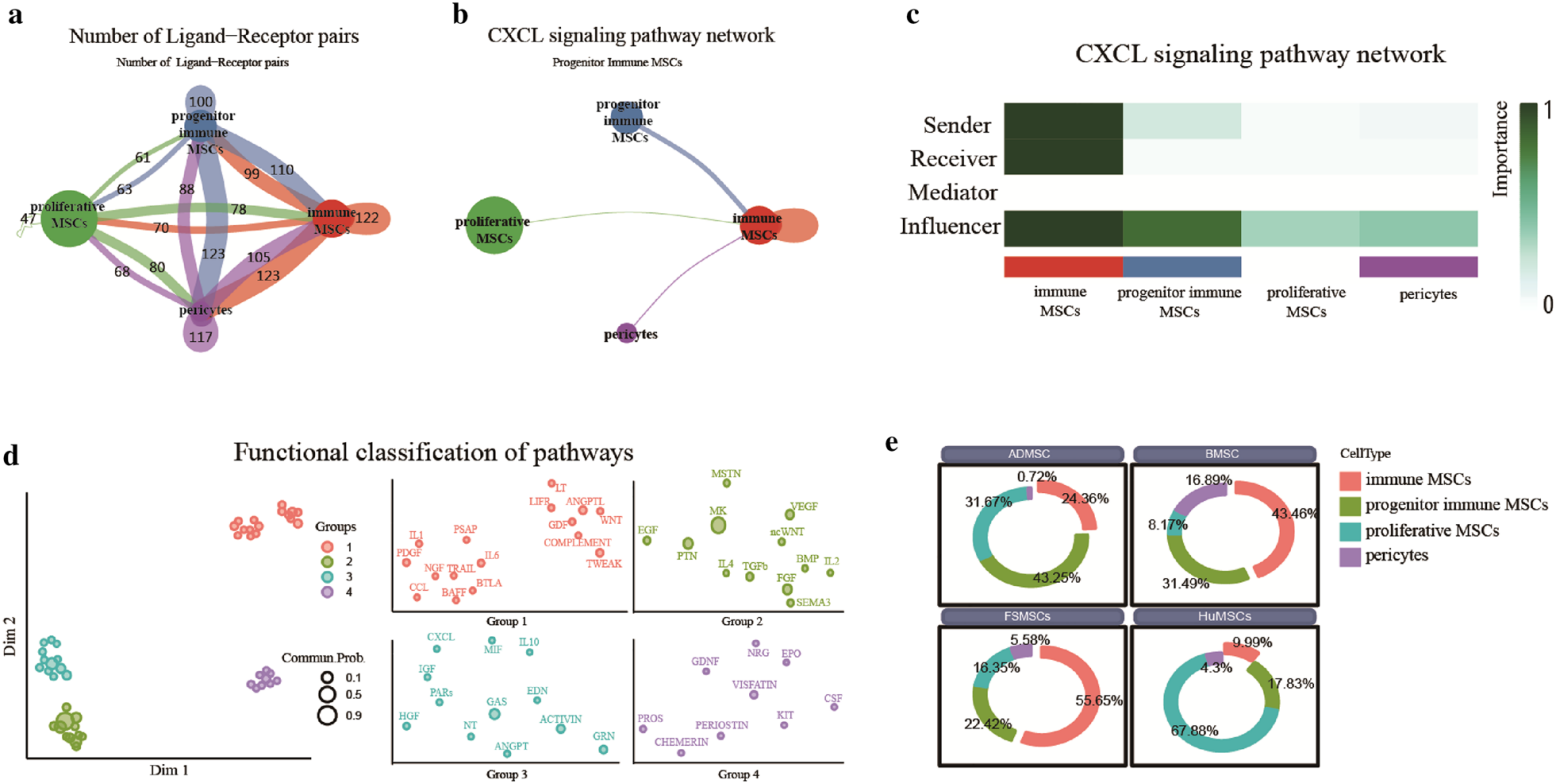
Results of cell–cell communication analysis and public database analysis. **a** Chord plot showing a total of 2309 ligand–receptor pairs between different MSC subsets. A higher number of ligand–receptor pairs is indicated by a thicker line. **b** Chord plot showing the expression of the CXCL signaling pathway between different MSC subsets. **c** Heatmap showing the sender, receiver, mediator, and influence scores between different MSC subsets. **d** The left plot shows the four signaling pathway groups with different colors. The right plots show all signaling pathways of specific pathway groups. A smaller dot indicates a higher probability of communication. **e** Circle plot showing the proportions of different MSC subsets among ADMSCs, BMSCs, FSMSCs and HuMSCs

We performed cell–cell communication analysis to explore the interactions among different MSC subsets. We identified a total of 2309 ligand–receptor pairs based on the integrated data (Fig. 6a). Moreover, these ligand–receptor pairs were mapped to 47 signaling pathways (Fig. 6d). We also observed the interaction of CXCL signaling pathways in all MSC subsets and determined the role of each cell subset at the signaling pathway level by calculating the network centrality index (Fig. 6b, c). Furthermore, the mediator score of immune MSCs was high, but the influence score was low, suggesting that immune MSCs were not a network node in the CXCL signaling pathway. However, both the sender and receiver scores of immune MSCs were high, indicating that immune MSCs played an autocrine role in the CXCL signaling pathway. In addition, we calculated the ligand–receptor pairs that contributed to the CXCL signaling pathway. *CXCL12-ACKR3* made the most significant contribution to the CXCL signaling pathway. This result suggests that *CXCL12-ACKR3* might play an autocrine role in immune MSCs. Finally, we quantified the functional similarity of all 47 signaling pathways and identified four signaling pathway groups (Fig. 6d).

### Public database analysis

The proportions of different MSC subsets were assessed among ADMSCs, BMSCs, FSMSCs and HuMSCs. Immune MSCs accounted for 24.36%, 43.46%, 55.65%, and 9.99% of cells among ADMSCs, BMSCs, FSMSCs, and HuMSCs respectively. Progenitor proliferative MSCs accounted for 43.25%, 31.49%, 22.42%, and 17.83% of ADMSCs, BMSCs, FSMSCs, and HuMSCs respectively. Proliferative MSCs accounted for 31.67%, 8.17%, 18.35%, and 67.88% of cells in ADMSCs, BMSCs, FSMSCs, and HuMSCs respectively. Pericyte accounted for 0.72%, 16.89%, 5.58%, and 4.3% of ADMSCs, BMSCs, FSMSCs, and HuMSCs respectively. (Fig. 6f).

## Discussion

MSCs were first isolated from bone marrow and can be used to treat immune-related diseases [2–8]. However, ethical disputes, the invasive operation required for collection, and the low yield of in vitro culture have limited the clinical application of MSCs derived from bone marrow. Thus, researchers have started to explore alternative sources. They have found that foreskin tissue and umbilical cord tissue are good substitutes for bone marrow due to the less-invasive nature of the collection procedure, their high clone proliferation potential in vitro, and their tremendous immunomodulatory abilities [10, 13]. In our study, we also identified FSMSCs and HuMSCs as MSCs according to the identification standard of the ISCT. The FSMSCs and HuMSCs were able to attach to plastic surfaces, and they had a typical long, spindle-shaped fibroblast-like morphology. Positive surface markers, such as CD73, CD90, and CD105, were expressed in more than 95% of the cells, while negative surface markers, such as CD45, CD34, CD11b, CD19, and HLA-DR, were expressed in less than 2% of the cells. The cells had osteogenic, adipogenic, and chondrogenic differentiation abilities.

The immunomodulatory properties of MSCs derived from different tissues are significantly heterogeneous, which is unfavorable for MSC applications [13]. However, a comparison of the immunomodulatory properties of FSMSCs and HuMSCs has not been reported. Thus, we compared the immunomodulatory properties of these cells in vitro. We found that FSMSCs and HuMSCs, similar to PBMCs, secreted inflammation-associated cytokines when they were cultured alone. There were no significant changes in the secretion of the proinflammatory cytokines IL-1β and TNF-α when LPS was used to stimulate the FSMSCs and HuMSCs. Moreover, FSMSCs and HuMSCs were able to reduce the secretion of proinflammatory cytokines and increase the secretion of anti-inflammatory cytokines when cocultured with LPS-prestimulated PBMCs. Nevertheless, FSMSCs stimulated PBMCs to secrete fewer proinflammatory cytokines and more anti-inflammatory cytokines than HuMSCs. This result suggested that FSMSCs showed more substantial immunomodulatory capacity than HuMSCs.

Some studies have reported that the heterogeneity of immunomodulatory capacity is significantly reduced and that the immunomodulatory capacity is notably enhanced when MSCs are pretreated with IFN-γ and/or TNF-α in vitro before application [51, 52]. These findings suggest that MSCs are plastic in immunomodulatory function and that they can differentiate into anti-inflammatory MSCs under these pretreatment conditions [52, 53]. However, this pretreatment also promotes the expression of immunogenicity-related genes in MSCs, which might accelerate the clearance of MSCs in vivo and enhance the potential risk of immune rejection, which is unfavorable for MSC application [51]. Recent evidence suggests that MSCs are mixed-cell populations consisting of different cell subsets with different biological functions [15–20]. This evidence indicates that specific MSC subsets may be able to be isolated and exhibit stable immunoregulatory ability without being limited by the heterogeneity of cells derived from different tissues. scRNA-seq can detect gene expression differences among cells and be used to explore different cell subsets [21]. Some studies have compared human umbilical cord mesenchymal stromal cells (HuMSCs) with MSCs derived from bone marrow [22], adipose tissue [23], and synovial fluid [12] through scRNA-seq, and the results reflect the heterogeneity of cell subsets of MSCs cultured in vitro. Therefore, we performed scRNA-seq to identify the potential MSC subset with more substantial immunomodulatory properties than other subsets and to explain the differences in immunomodulatory abilities of MSCs derived from different tissues.

We performed scRNA-seq on FSMSCs and HuMSCs and finally obtained 7335 and 12,542 cells after quality control. In addition, we clustered and annotated FSMSCs and HuMSCs according to the surface markers of MSCs (CD73+, CD90+, CD105+, CD45−, CD34−, CD11b−, CD19−, and HLA-DR−) [1]. Only 93.05% of FSMSCs were MSCs, while 95.85% of HuMSCs were MSCs. Then, we integrated the FSMSCs and HuMSCs annotated as MSCs, and four cell subsets were identified: proliferative MSCs (*MKI67*^+^, *CD146*^*low*+^, *NG2*^+^, *PDGFRB*^*−*^), pericytes (*CD146*^*high*+^, *PDGFRB*^+^, *MKI67*^*−*^, *CD31*^*−*^, *CD45*^*−*^, *CD34*^*−*^), immune MSCs (*CXCL12*^*high*+^, *PTGIS*^*high*+^, *PDGFRB*^+^, *CD146*^*−*^, *MKI67*^*−*^) and progenitor proliferative MSCs (*CXCL12*^*low*+^, *PTGIS*^*low*+^, *PDGFRB*^+^, *CD146*^*−*^, *MKI67*^*−*^). This analysis was also crucial for selecting the MSC subset used in regenerative processes.

We inferred the differentiation degrees of the four MSC subsets with the R package CytoTRACE. We found that the differentiation degrees of proliferative MSCs and progenitor proliferative MSCs were higher than those of immune MSCs and pericytes. Some studies have reported that MSCs might originate from pericytes [50]. Therefore, we defined pericytes as the starting point of the differentiation trajectory and built two differentiation trajectories of MSCs based on transcriptional similarity with the R package slingshot. In the first trajectory, the cells started from pericytes and differentiated into proliferative MSCs. However, in the second trajectory, the cells started from pericytes, passed through the immune MSC and progenitor proliferative MSC stages, and finally differentiated into proliferative MSCs. This finding reveals that progenitor proliferative MSCs might be a transitional stage from immune MSCs to proliferative MSCs. Proliferative MSCs were at the ends of two differentiation trajectories, and proliferative MSCs expressed lower levels of *CXCL12* and *PTGIS* than immune MSCs and pericytes. This result indicates that the immunomodulatory properties of MSCs might be influenced by the degree of differentiation. In addition, we found that the transcriptional activity of immune MSCs was intense according to RNA velocity analysis, while the expression of *MKI67* in immune MSCs was lower than that in other subsets. This result suggested that immune MSCs showed weaker proliferative activity but still expressed many genes to maintain their biological function.

When we compared the gene expression between FSMSCs and HuMSCs, we obtained 37 immune-related differentially expressed genes and divided them into seven immune categories. Among them, *RARRES2*, *B2M*, *LGALS3*, and *ADM*, which were highly expressed in FSMSCs, belonged to the “Antimicrobials” category and have been reported to possess immunomodulatory properties and antimicrobial ability [54–57]. This result indicates that FSMSCs might have more substantial immunomodulatory properties and antimicrobial ability than HuMSCs. Furthermore, we found that a variety of immune-related differentially expressed genes, gene sets, and regulons were enriched in immune MSCs. When we compared the gene expression among different MSC subsets, we also identified 14 immune-related differentially expressed genes. *BIRC5* and *PBK* were downregulated in immune MSCs, while *CXCL12*, *TNFRSF11B*, *ADM*, *LGALS3*, *NUPR1*, *PTGIS*, *IGFBP4 FGF7*, *B2M*, *CRLF1*, *ACKR*4, and *SERPINF* were upregulated. *B2M*, *LGALS3*, and *ADM* belonged to the “Antimicrobials” category and have been reported to possess immunomodulatory properties and antimicrobial ability [55–57]. This result indicates that immune MSCs also have greater immunomodulatory capability than other MSC subsets. Moreover, the immune-related differentially expressed gene sets “IL6 JAK STAT3 SIGNALING”, “INTERFERON GAMMA RESPONSE”, and “INTERFERON ALPHA RESPONSE” and the hypoxia-related differentially expressed gene set “HYPOXIA” were significantly enriched in immune MSCs when we compared the enrichment of hallmark gene sets among the four MSC subsets. In previous studies, hypoxia has been shown to promote the immunoregulatory capacity of MSCs [58]. This result indicates that immune MSCs might possess greater immunomodulatory capacity than the other cell subsets.

Moreover, we constructed a gene regulatory network and identified 22 CTSRs in immune MSCs. Among them, the immune-related regulons *IRF2* and *IRF4*, which were associated with interferon regulation in response to viral infection and the regulation of interferon-inducible genes, were highly expressed in immune MSCs and might regulate the expression of the target gene *CXCL12* based on the single-cell RNA-seq data. *IRF2* and *IRF4* belong to the interferon regulatory factor family. Usually, members of this family are lymphocyte-specific and can negatively regulate Toll-like receptor signaling. Negative regulation is essential for the activation of innate and adaptive immunity. Some studies have reported that *IRF4* is an inhibitor of TLR-induced inflammatory pathways [59]. This indicates that the immune-related regulons *IRF2* and *IRF4* play a significant role in maintaining biological function.

Then, a total of 2309 ligand–receptor pairs were identified and mapped to 47 signaling pathways through cell–cell communication analysis. We also quantified the functional similarity of all 47 signaling pathways and identified four signaling pathway groups. The results indicated that the immune MSC subset was close to the other cell subsets. We found that *CXCL12–ACKR3* made the most significant contribution to the CXCL signaling pathway and might play an autocrine role in immune MSCs. *ACKR3* is the scavenger receptor of *CXCL12*, which can regulate the concentration gradient of *CXCL12* [60]. The expression of *ACKR3* in immune MSCs can prevent excessive *CXCL12* from causing damage to the cells themselves. Some studies have found that blocking *ACKR3* with the antagonist AMD3100 can improve the immunosuppressive tumor microenvironment [61]. This suggests that *ACKR3* plays an essential role in regulating the concentration gradient of CXCL12 and maintains the immunosuppressive function. Therefore, *CXCL12-ACKR3* might play a vital role in immune MSCs. Finally, we quantified the functional similarity of all 47 signaling pathways and identified four signaling pathway groups. CXCL, IGF, MIF, IL10, PARs, HGF, NT, GAS, EDN, ACTIVIN, and GRN belonged to the same signaling pathway group, which implies that the related signaling pathways might help the CXCL pathway maintain the biological functions of immune MSCs.

In addition, we compared the cell subset compositions of MSCs derived from different tissues. Immune MSCs accounted for 24.36%, 43.46%, 55.65%, and 9.99% of cells among ADMSCs, BMSCs, FSMSCs, and HuMSCs, respectively. This result suggests that foreskin tissue might be an ideal source for isolating immune MSCs. We believe that MSCs are susceptible to external environmental influences. Moreover, foreskin tissue is frequently exposed to bacterial and viral attacks due to its specific environment. Furthermore, foreskin tissue is prone to ischemia or hypoxia because of insufficient blood supply. Hypoxia plays an important role in the immunomodulatory capacity of MSCs [58]. Thus, MSCs derived from foreskin tissue are more likely than MSCs derived from other tissues to differentiate into anti-inflammatory MSCs.

In summary, there was significant heterogeneity in the immunomodulatory properties of MSCs derived from different tissues. Furthermore, FSMSCs showed better immunomodulatory capacity than HuMSCs in vitro. Immune MSCs may be the fundamental cause of the heterogeneity of immunoregulatory properties revealed through scRNA-seq. Significantly, our study was limited to in vitro work. However, it still provides new insights to explain the immunomodulatory heterogeneity of MSCs and indicates that immune MSCs can be isolated to exert stable immunoregulatory ability without being limited by the heterogeneity of MSCs derived from different tissues.

## Conclusions

FSMSCs and HuMSCs were successfully identified as MSCs. Moreover, FSMSCs showed better immunomodulatory capacity than HuMSCs in vitro. Immune MSCs may play a vital role in the difference in immunoregulatory properties given the results of bioinformatics analysis based on scRNA-seq data. This study provides new insights suggesting that immune MSCs can be isolated to exert stable immunoregulatory functions without being limited by the heterogeneity of MSCs derived from different tissues.

## Supplementary Information

**Additional file 1.** The statistical result of PBMCs/HuMSCs/FSMSCs cultured alone: **Table S1.** The result of Kruskal–Wallis rank sum test between different groups at special time point. **Table S2.** The result of pairwise comparisons in different time points. **Table S3.** The result of Kruskal–Wallis rank sum test between different time points at special group. **Table S4.** The result of pairwise comparisons in different groups.

**Additional file 2.** The statistical result of PBMCs/HuMSCs/FSMSCs stimulated by LPS: **Table S5.** The result of Kruskal–Wallis rank sum test between different groups at special time point. **Table S6.** The result of pairwise comparisons in different time points. **Table S7.** The result of Kruskal–Wallis rank sum test between different time points at special group. **Table S8.** The result of pairwise comparisons in different groups.

**Additional file 3.** The statistical result of HuMSCs/FSMSCs cocultured with PBMCs without stimulation: **Table S9.** The result of Kruskal–Wallis rank sum test between different groups at special time point. **Table S10.** The result of pairwise comparisons in different time points. **Table S11.** The result of Kruskal–Wallis rank sum test between different time points at special group. **Table S12.** The result of pairwise comparisons in different groups.

**Additional file 4.** The statistical results of HuMSCs/FSMSCs cocultured with LPS pretreated PBMCs: **Table S13.** The result of Kruskal–Wallis rank sum test between different groups at special time point. **Table S14.** The result of pairwise comparisons in different time points. **Table S15.** The result of Kruskal–Wallis rank sum test between different time points at special group. **Table S16.** The result of pairwise comparisons in different groups.

**Additional file 5.** The table of the immune-related gene.

**Additional file 6.** The enrichment score of the hallmark gene set of different MSC subsets.

**Additional file 7.** The adjusted p value of the hallmark gene set of different MSC subsets.

**Additional file 8.** The differentially expressed gene between FSMSCs and HuMSCs.

**Additional file 9.** The differentially expressed gene between different MSC subsets.

**Additional file 10.** The percentage of the variance of each PC in the population variance.

**Additional file 11.** The evaluation result of the first 50 PCs of FSMSCs: **Plot S1.** The JackStrawPlot of FSMSCs. **Plot S2.** The ElbowPlot of FSMSCs.

**Additional file 12.** The evaluation result of the first 50 PCs of HuMSCs: **Plot S1.** The JackStrawPlot of HuMSCs. **Plot S2.** The ElbowPlot of HuMSCs.

**Additional file 13.** The evaluation result of the first 50 PCs of the integrated data: **Plot S1.** The JackStrawPlot of the integrated data. **Plot S2.** The ElbowPlot of the integrated data.

## Data Availability

Additional tables and high-resolution plots are included in Additional files. The scRNA-seq data have been shared in Mendeley Data (https://data.mendeley.com/datasets/f4b2ykfv56/1).

## Abbreviations

ADMSCs: Adipose mesenchymal stromal cells
BMSCs: Bone marrow mesenchymal stromal cells
CTSRs: Cell-type-specific regulons
DMEM/F12: Dulbecco’s modified Eagle medium/nutrient mixture F-12
ELISA: Enzyme-linked immunosorbent assay
FSMSCs: Foreskin mesenchymal stromal cells
GSEA: Gene set enrichment analysis
HuMSCs: Human umbilical cord mesenchymal stromal cells
ISCT: International Association for Cell Therapy
Log2FC: Log2(fold change)
LPS: Lipopolysaccharide
MSCs: Mesenchymal stromal cells
PBMC: Peripheral blood mononuclear cells
PBS: Phosphate buffer solution
PCA: Principal component analysis
PCs: Principal components
RAS: Regulon activity score
RSS: Regulon specific score
scRNA-seq: Single-cell RNA sequencing
TSNE: T-distributed stochastic neighbor embedding
UMAP: Uniform manifold approximation and projection
