# Supplementary material for: Single-cell RNA sequencing reveals the potential mechanism of heterogeneity of immunomodulatory properties of foreskin and umbilical cord mesenchymal stromal cells
Source: Cell Biosci. 2022 Jul 22;12:115. doi: 10.1186/s13578-022-00848-w (PMC9306236; doi:10.1186/s13578-022-00848-w)
Supplement: Supplementary file 1 — Additional file 1. The statistical result of PBMCs/HuMSCs/FSMSCs cultured alone: Table S1. The result of Kruskal–Wallis rank sum test between different groups at special time point. Table S2. The result of pairwise comparisons in different time points. Table S3. The result of Kruskal–Wallis rank sum test between different time points at special group. Table S4. The result of pairwise comparisons in different groups. [file 13578_2022_848_MOESM1_ESM.docx]

| Table S1: The Result of Kruskal-Wallis Rank Sum Test between Different Groups at Special Time Point | | | | |
| --- | --- | --- | --- | --- |
| Cytokine | Time | | | |
|  | 2h | 4h | 12h | 24h |
| IL-1β | 0.30 | 0.05 | 0.29 | 0.19 |
| IL-6 | 0.03 | 0.03 | 0.02 | 0.03 |
| IL-10 | 0.17 | 0.15 | 0.48 | 0.67 |
| TNF-α | 0.03 | 0.04 | 0.03 | 0.06 |
| TGF-β1 | 0.06 | 0.06 | 0.03 | 0.07 |
| The p value of Kruskal-Wallis Rank Sum Test shows whether the difference of cytokine concentration between different groups (FSMSCs/HuMSCs/PBMCs) is statistically significant at special time point. See the Table 2 for all pairwise comparisons in detail. | | | | |

| Table S2: The Result of Pairwise Comparisons in Different Time Points | | | | | |
| --- | --- | --- | --- | --- | --- |
| Comparison | Cytokine | 2h | 4h | 12h | 24h |
| FSMSCs - HuMSCs | IL-1β | 1.00 | 0.30 | 1.00 | 1.00 |
| FSMSCs - PBMCs | IL-1β | 0.54 | 0.05 | 0.70 | 0.30 |
| HuMSCs - PBMCs | IL-1β | 0.54 | 1.00 | 0.41 | 0.41 |
| FSMSCs - HuMSCs | IL-6 | 0.54 | 0.54 | 0.52 | 0.54 |
| FSMSCs - PBMCs | IL-6 | 0.54 | 0.54 | 0.52 | 0.54 |
| HuMSCs - PBMCs | IL-6 | 0.02 | 0.02 | 0.02 | 0.02 |
| FSMSCs - HuMSCs | IL-10 | 1.00 | 1.00 | 1.00 | 1.00 |
| FSMSCs - PBMCs | IL-10 | 0.21 | 0.26 | 0.68 | 1.00 |
| HuMSCs - PBMCs | IL-10 | 0.52 | 0.30 | 1.00 | 1.00 |
| FSMSCs - HuMSCs | TNF-α | 0.54 | 0.89 | 0.54 | 1.00 |
| FSMSCs - PBMCs | TNF-α | 0.02 | 0.41 | 0.02 | 0.08 |
| HuMSCs - PBMCs | TNF-α | 0.54 | 0.03 | 0.54 | 0.22 |
| FSMSCs - HuMSCs | TGF-β1 | 1.00 | 1.00 | 0.54 | 0.16 |
| FSMSCs - PBMCs | TGF-β1 | 0.22 | 0.08 | 0.54 | 1.00 |
| HuMSCs - PBMCs | TGF-β1 | 0.08 | 0.22 | 0.02 | 0.11 |
| The p values of multiple-comparisons are calculated by Dunn's Test | | | | | |

| Table S3: The Result of Kruskal-Wallis Rank Sum Test between Different Time Points at Special Group | | | |
| --- | --- | --- | --- |
| Cytokine | Group | | |
|  | FSMSCs | HuMSCs | PBMCs |
| IL-1β | 0.15 | 0.08 | 0.09 |
| IL-6 | 0.02 | 0.01 | 0.03 |
| IL-10 | 0.59 | 0.16 | 0.45 |
| TNF-α | 0.06 | 0.11 | 0.74 |
| TGF-β1 | 0.02 | 0.02 | 0.02 |
| The p value of Kruskal-Wallis Rank Sum Test shows whether the difference of cytokine concentration between different time points (2h/4h/12h/24h) is statistically significant at special group. See the Table 4 for all pairwise comparisons in detail. | | | |

| Table S4: The Result of Pairwise Comparisons in Different Groups | | | | |
| --- | --- | --- | --- | --- |
| Comparison | Cytokine | FSMSCs | HuMSCs | PBMCs |
| 12h - 2h | IL-1β | 1.00 | 0.25 | 0.42 |
| 12h - 24h | IL-1β | 1.00 | 1.00 | 1.00 |
| 12h - 4h | IL-1β | 0.54 | 1.00 | 1.00 |
| 2h - 24h | IL-1β | 1.00 | 0.10 | 0.14 |
| 2h - 4h | IL-1β | 0.19 | 0.54 | 0.25 |
| 24h - 4h | IL-1β | 1.00 | 1.00 | 1.00 |
| 12h - 2h | IL-6 | 0.25 | 0.24 | 0.68 |
| 12h - 24h | IL-6 | 1.00 | 1.00 | 1.00 |
| 12h - 4h | IL-6 | 1.00 | 1.00 | 1.00 |
| 2h - 24h | IL-6 | 0.01 | 0.01 | 0.68 |
| 2h - 4h | IL-6 | 1.00 | 1.00 | 0.02 |
| 24h - 4h | IL-6 | 0.25 | 0.24 | 1.00 |
| 12h - 2h | IL-10 | 1.00 | 0.42 | 1.00 |
| 12h - 24h | IL-10 | 1.00 | 1.00 | 1.00 |
| 12h - 4h | IL-10 | 1.00 | 0.53 | 1.00 |
| 2h - 24h | IL-10 | 1.00 | 0.84 | 0.75 |
| 2h - 4h | IL-10 | 1.00 | 1.00 | 1.00 |
| 24h - 4h | IL-10 | 1.00 | 1.00 | 1.00 |
| 12h - 2h | TNF-α | 1.00 | 1.00 | 1.00 |
| 12h - 24h | TNF-α | 0.54 | 0.85 | 1.00 |
| 12h - 4h | TNF-α | 1.00 | 0.33 | 1.00 |
| 2h - 24h | TNF-α | 1.00 | 0.85 | 1.00 |
| 2h - 4h | TNF-α | 0.33 | 0.33 | 1.00 |
| 24h - 4h | TNF-α | 0.08 | 1.00 | 1.00 |
| 12h - 2h | TGF-β1 | 1.00 | 1.00 | 0.85 |
| 12h - 24h | TGF-β1 | 0.33 | 0.25 | 0.08 |
| 12h - 4h | TGF-β1 | 1.00 | 0.01 | 1.00 |
| 2h - 24h | TGF-β1 | 1.00 | 1.00 | 1.00 |
| 2h - 4h | TGF-β1 | 0.33 | 0.25 | 0.68 |
| 24h - 4h | TGF-β1 | 0.01 | 1.00 | 0.06 |
| The p values of multiple-comparisons are calculated by Dunn's Test | | | | |
