# Supplementary material for: Single-cell RNA sequencing reveals the potential mechanism of heterogeneity of immunomodulatory properties of foreskin and umbilical cord mesenchymal stromal cells
Source: Cell Biosci. 2022 Jul 22;12:115. doi: 10.1186/s13578-022-00848-w (PMC9306236; doi:10.1186/s13578-022-00848-w)
Supplement: Supplementary file 3 — Additional file 3. The statistical result of HuMSCs/FSMSCs cocultured with PBMCs without stimulation: Table S9. The result of Kruskal–Wallis rank sum test between different groups at special time point. Table S10. The result of pairwise comparisons in different time points. Table S11. The result of Kruskal–Wallis rank sum test between different time points at special group. Table S12. The result of pairwise comparisons in different groups. [file 13578_2022_848_MOESM3_ESM.docx]

| Table S9: The Result of Kruskal-Wallis Rank Sum Test between Different Groups at Special Time Point | | | | |
| --- | --- | --- | --- | --- |
| Cytokine | Time | | | |
|  | 2h | 4h | 12h | 24h |
| IL-1β | 0.07 | 0.06 | 0.06 | 0.04 |
| IL-6 | 0.03 | 0.03 | 0.03 | 0.03 |
| IL-10 | 0.08 | 0.04 | 0.03 | 0.06 |
| TNF-α | 0.05 | 0.03 | 0.03 | 0.03 |
| TGF-β1 | 0.03 | 0.03 | 0.03 | 0.03 |
| The p value of Kruskal-Wallis Rank Sum Test shows whether the difference of cytokine concentration between different groups (FSMSCs/HuMSCs/PBMCs) is statistically significant at special time point. See the Table 2 for all pairwise comparisons in detail. | | | | |

| Table S10: The Result of Pairwise Comparisons in Different Time Points | | | | | |
| --- | --- | --- | --- | --- | --- |
| Comparison | Cytokine | 2h | 4h | 12h | 24h |
| FSMSCs+PBMCs - HuMSCs+PBMCs | IL-1β | 0.16 | 0.08 | 1.00 | 0.88 |
| FSMSCs+PBMCs - PBMCs | IL-1β | 1.00 | 0.22 | 0.11 | 0.03 |
| HuMSCs+PBMCs - PBMCs | IL-1β | 0.11 | 1.00 | 0.15 | 0.40 |
| FSMSCs+PBMCs - HuMSCs+PBMCs | IL-6 | 0.53 | 0.54 | 0.54 | 0.54 |
| FSMSCs+PBMCs - PBMCs | IL-6 | 0.02 | 0.02 | 0.02 | 0.02 |
| HuMSCs+PBMCs - PBMCs | IL-6 | 0.53 | 0.54 | 0.54 | 0.54 |
| FSMSCs+PBMCs - HuMSCs+PBMCs | IL-10 | 0.54 | 0.89 | 0.54 | 0.08 |
| FSMSCs+PBMCs - PBMCs | IL-10 | 0.08 | 0.03 | 0.02 | 0.22 |
| HuMSCs+PBMCs - PBMCs | IL-10 | 1.00 | 0.41 | 0.54 | 1.00 |
| FSMSCs+PBMCs - HuMSCs+PBMCs | TNF-α | 0.05 | 0.02 | 0.02 | 0.02 |
| FSMSCs+PBMCs - PBMCs | TNF-α | 1.00 | 0.54 | 0.54 | 0.54 |
| HuMSCs+PBMCs - PBMCs | TNF-α | 0.30 | 0.54 | 0.54 | 0.54 |
| FSMSCs+PBMCs - HuMSCs+PBMCs | TGF-β1 | 0.54 | 0.54 | 0.54 | 0.02 |
| FSMSCs+PBMCs - PBMCs | TGF-β1 | 0.02 | 0.02 | 0.02 | 0.54 |
| HuMSCs+PBMCs - PBMCs | TGF-β1 | 0.54 | 0.54 | 0.54 | 0.54 |
| The p values of multiple-comparisons are calculated by Dunn's Test | | | | | |

| Table S11: The Result of Kruskal-Wallis Rank Sum Test between Different Time Points at Special Group | | | |
| --- | --- | --- | --- |
| Cytokine | Group | | |
|  | FSMSCs+PBMCs | HuMSCs+PBMCs | PBMCs |
| IL-1β | 0.25 | 0.03 | 0.09 |
| IL-6 | 0.02 | 0.02 | 0.03 |
| IL-10 | 0.02 | 0.64 | 0.45 |
| TNF-α | 0.02 | 0.08 | 0.74 |
| TGF-β1 | 0.02 | 0.02 | 0.02 |
| The p value of Kruskal-Wallis Rank Sum Test shows whether the difference of cytokine concentration between different time points (2h/4h/12h/24h) is statistically significant at special group. See the Table 4 for all pairwise comparisons in detail. | | | |

| Table S12: The Result of Pairwise Comparisons in Different Groups | | | | |
| --- | --- | --- | --- | --- |
| Comparison | Cytokine | FSMSCs+PBMCs | HuMSCs+PBMCs | PBMCs |
| 12h - 2h | IL-1β | 1.00 | 0.67 | 0.42 |
| 12h - 24h | IL-1β | 1.00 | 1.00 | 1.00 |
| 12h - 4h | IL-1β | 1.00 | 0.32 | 1.00 |
| 2h - 24h | IL-1β | 0.28 | 0.19 | 0.14 |
| 2h - 4h | IL-1β | 1.00 | 1.00 | 0.25 |
| 24h - 4h | IL-1β | 1.00 | 0.07 | 1.00 |
| 12h - 2h | IL-6 | 0.25 | 0.25 | 0.68 |
| 12h - 24h | IL-6 | 1.00 | 1.00 | 1.00 |
| 12h - 4h | IL-6 | 1.00 | 1.00 | 1.00 |
| 2h - 24h | IL-6 | 0.01 | 0.01 | 0.68 |
| 2h - 4h | IL-6 | 1.00 | 1.00 | 0.02 |
| 24h - 4h | IL-6 | 0.25 | 0.25 | 1.00 |
| 12h - 2h | IL-10 | 0.42 | 1.00 | 1.00 |
| 12h - 24h | IL-10 | 1.00 | 1.00 | 1.00 |
| 12h - 4h | IL-10 | 1.00 | 1.00 | 1.00 |
| 2h - 24h | IL-10 | 0.03 | 1.00 | 0.75 |
| 2h - 4h | IL-10 | 1.00 | 1.00 | 1.00 |
| 24h - 4h | IL-10 | 0.14 | 1.00 | 1.00 |
| 12h - 2h | TNF-α | 0.02 | 1.00 | 1.00 |
| 12h - 24h | TNF-α | 1.00 | 1.00 | 1.00 |
| 12h - 4h | TNF-α | 0.33 | 0.14 | 1.00 |
| 2h - 24h | TNF-α | 0.19 | 1.00 | 1.00 |
| 2h - 4h | TNF-α | 1.00 | 1.00 | 1.00 |
| 24h - 4h | TNF-α | 1.00 | 0.25 | 1.00 |
| 12h - 2h | TGF-β1 | 1.00 | 0.25 | 0.85 |
| 12h - 24h | TGF-β1 | 0.25 | 1.00 | 0.08 |
| 12h - 4h | TGF-β1 | 1.00 | 1.00 | 1.00 |
| 2h - 24h | TGF-β1 | 0.01 | 1.00 | 1.00 |
| 2h - 4h | TGF-β1 | 0.25 | 0.01 | 0.68 |
| 24h - 4h | TGF-β1 | 1.00 | 0.25 | 0.06 |
| The p values of multiple-comparisons are calculated by Dunn's Test | | | | |
