# Supplementary figures and images for: Single-cell RNA sequencing reveals the potential mechanism of heterogeneity of immunomodulatory properties of foreskin and umbilical cord mesenchymal stromal cells
Source: Cell Biosci. 2022 Jul 22;12:115. doi: 10.1186/s13578-022-00848-w (PMC9306236; doi:10.1186/s13578-022-00848-w)

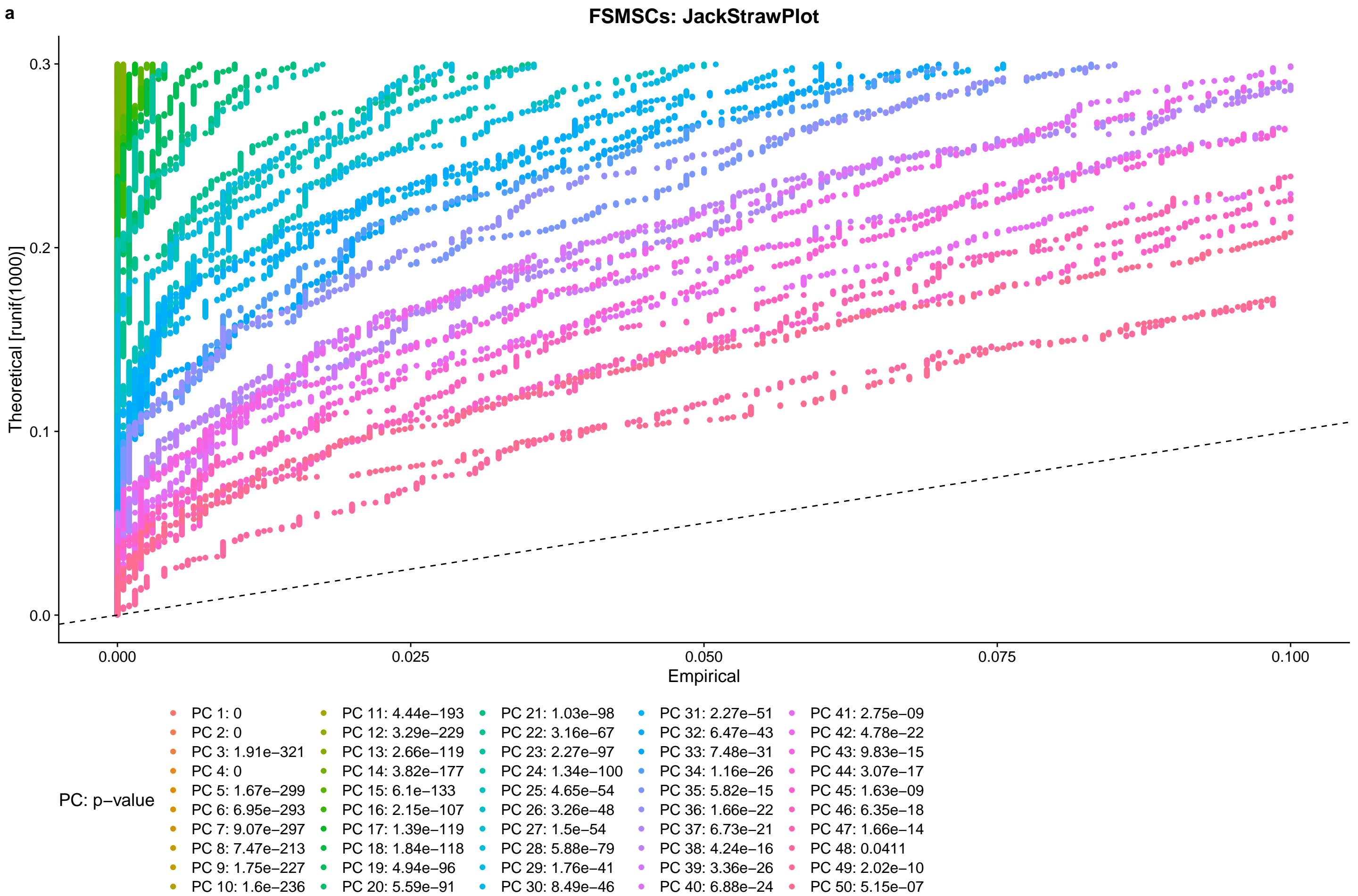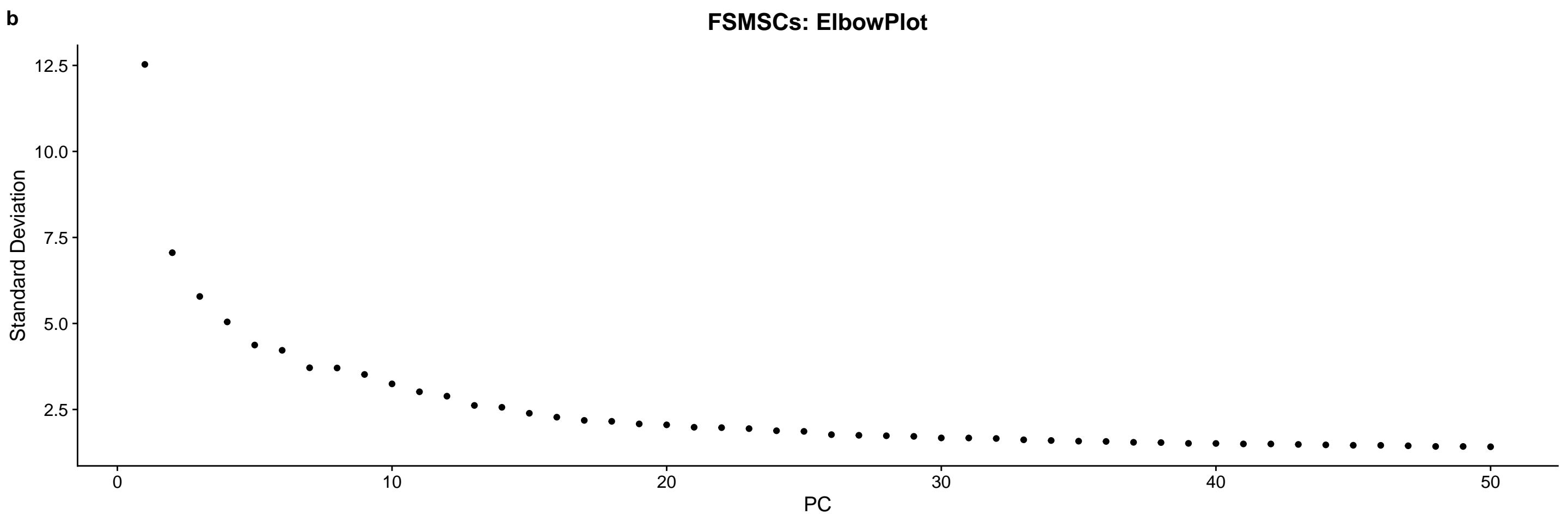

Supplement: Supplementary file 11 — Additional file 11. The evaluation result of the first 50 PCs of FSMSCs: Plot S1. The JackStrawPlot of FSMSCs. Plot S2. The ElbowPlot of FSMSCs. [file 13578_2022_848_MOESM11_ESM.pdf]

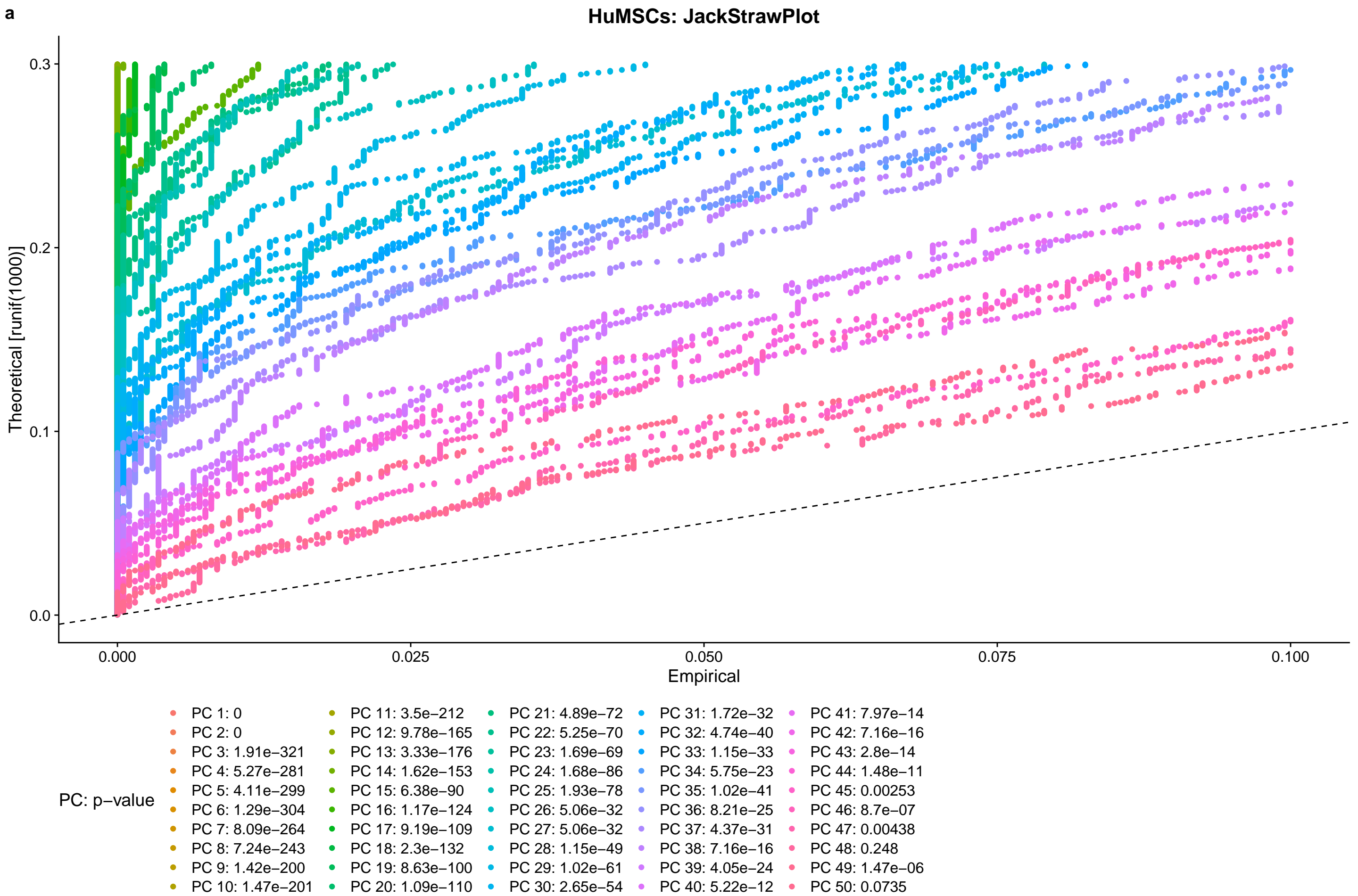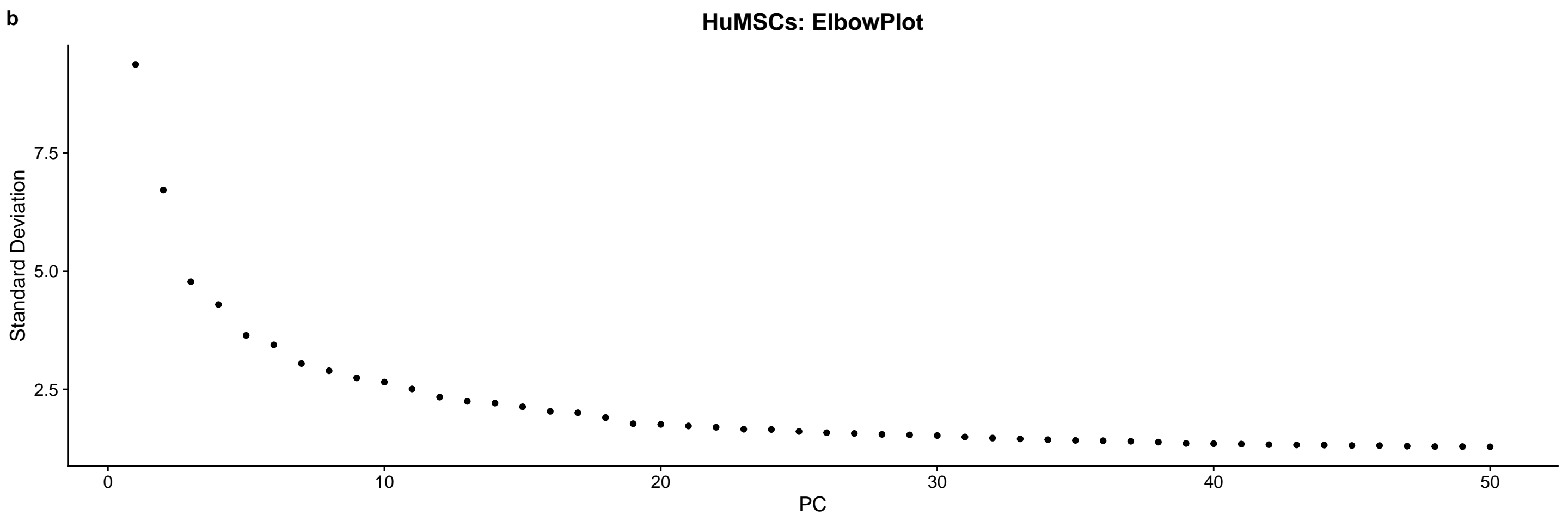

Supplement: Supplementary file 12 — Additional file 12. The evaluation result of the first 50 PCs of HuMSCs: Plot S1. The JackStrawPlot of HuMSCs. Plot S2. The ElbowPlot of HuMSCs. [file 13578_2022_848_MOESM12_ESM.pdf]

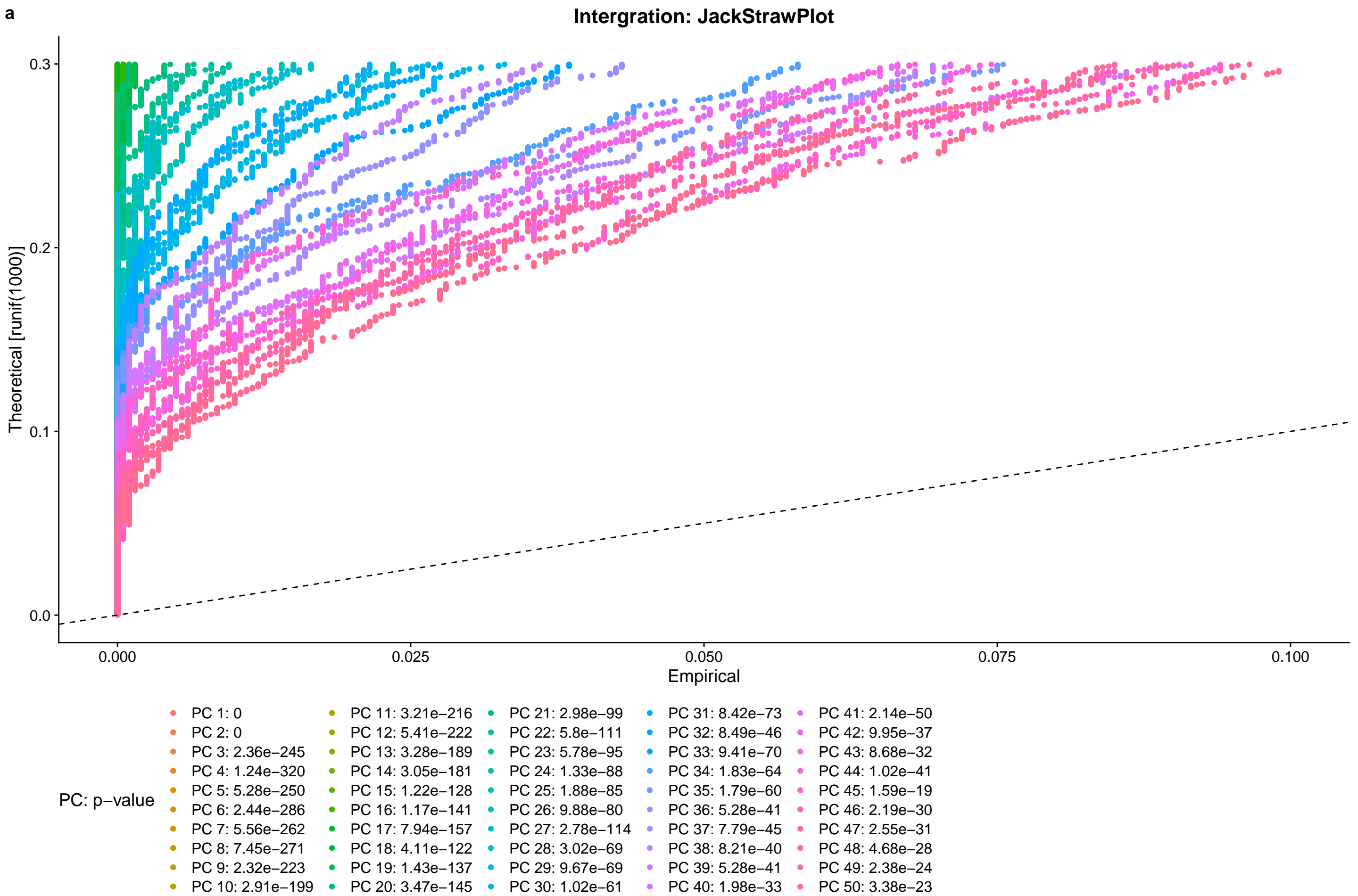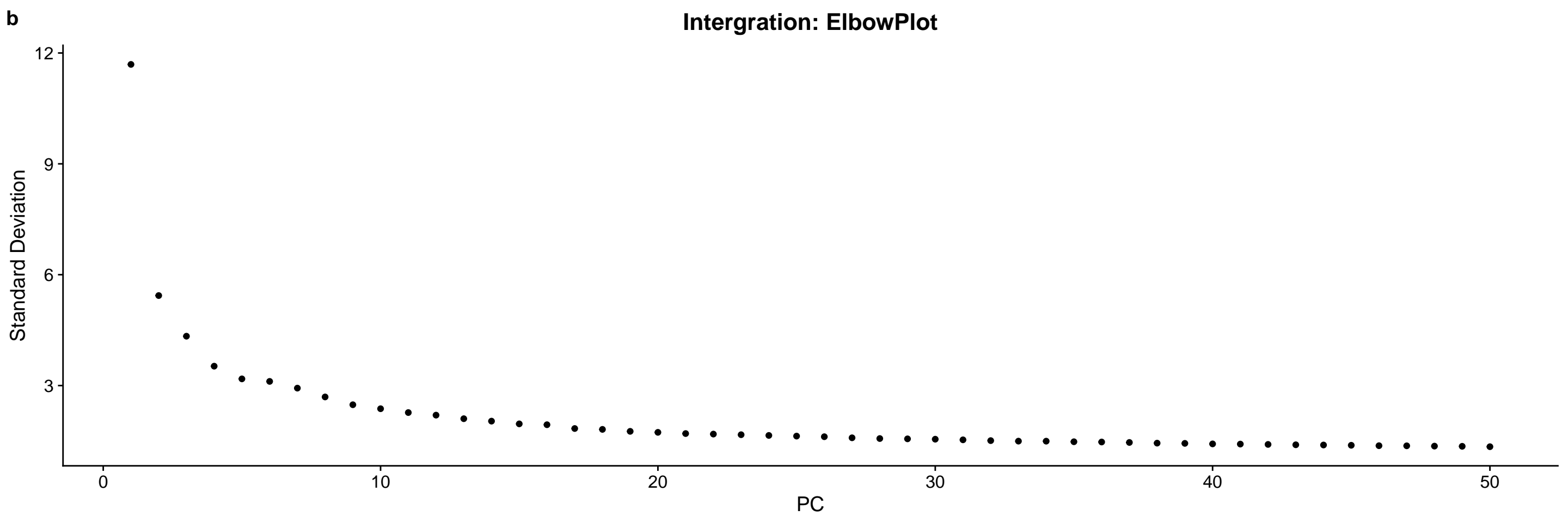

Supplement: Supplementary file 13 — Additional file 13. The evaluation result of the first 50 PCs of the integrated data: Plot S1. The JackStrawPlot of the integrated data. Plot S2. The ElbowPlot of the integrated data. [file 13578_2022_848_MOESM13_ESM.pdf]
